# Quantification of HTLV-1 Clonality and TCR Diversity

**DOI:** 10.1371/journal.pcbi.1003646

**Published:** 2014-06-19

**Authors:** Daniel J. Laydon, Anat Melamed, Aaron Sim, Nicolas A. Gillet, Kathleen Sim, Sam Darko, J. Simon Kroll, Daniel C. Douek, David A. Price, Charles R. M. Bangham, Becca Asquith

**Affiliations:** 1 Section of Immunology, Wright-Fleming Institute, Imperial College School of Medicine, London, United Kingdom; 2 Centre for Integrative Systems Biology and Bioinformatics, South Kensington Campus, Imperial College, London, United Kingdom; 3 Department of Molecular and Cellular Epigenetics, University of Liège, Liège, Belgium; 4 Section of Paediatrics, Wright-Fleming Institute, Imperial College School of Medicine, London, United Kingdom; 5 Vaccine Research Center, National Institutes of Health, Bethesda, Maryland, United States of America; 6 Institute of Infection and Immunity, Cardiff University School of Medicine, Cardiff, Wales, United Kingdom; La Jolla Institute for Allergy and Immunology, United States of America

## Abstract

Estimation of immunological and microbiological diversity is vital to our understanding of infection and the immune response. For instance, what is the diversity of the T cell repertoire? These questions are partially addressed by high-throughput sequencing techniques that enable identification of immunological and microbiological “species” in a sample. Estimators of the number of unseen species are needed to estimate population diversity from sample diversity. Here we test five widely used non-parametric estimators, and develop and validate a novel method, *DivE*, to estimate species richness and distribution. We used three independent datasets: (i) viral populations from subjects infected with human T-lymphotropic virus type 1; (ii) T cell antigen receptor clonotype repertoires; and (iii) microbial data from infant faecal samples. When applied to datasets with rarefaction curves that did not plateau, existing estimators systematically increased with sample size. In contrast, *DivE* consistently and accurately estimated diversity for all datasets. We identify conditions that limit the application of *DivE*. We also show that *DivE* can be used to accurately estimate the underlying population frequency distribution. We have developed a novel method that is significantly more accurate than commonly used biodiversity estimators in microbiological and immunological populations.

## Author Summary

The “unseen species problem” is ubiquitous in biology and is frequently encountered outside its original setting in population ecology. For example, the human retrovirus HTLV-1 persists within hosts in multiple, genetically identical clones of infected cells. However, the number of clones in one host is unknown; this knowledge is required for an understanding of how the virus survives despite a strong host immune response. The problem arises again in estimating the diversity of the T-cell repertoire, which influences adaptive immunity. For example, the T-cell diversity may influence the outcome of viral challenge. While there have been numerous attempts to address the unseen species problem, there is currently no consensus on how to do so in immunology and microbiology. The aim of this study was to identify a suitable method to estimate the number of species in immunological and microbiological populations. We found that five existing estimators we tested performed poorly across three data sources (HTLV-1 clonality, T cell receptor, and microbial data). We therefore developed a new estimator, *DivE*, which significantly outperformed the other estimators. Accurate diversity quantification allows better evaluation of the impact on immunity from factors such as ageing and infection.

## Introduction

How can we estimate diversity from a population sample? In viral infections, the number of viral variants and their population structure inform our understanding of disease pathogenesis, and can suggest treatment strategies [1], [2]. In immunology, the repertoire and population structure of B cell and T cell receptor clonotypes vary with age [3]–[7], and are intimately linked to antimicrobial protective efficacy. In the human microbiome, decreased diversity of the gastrointestinal microbiota is associated with atopy [8], Crohn's disease and ulcerative colitis [9], [10].

A complete census is usually impossible and so estimators of the number of unseen “species” are required. Here we use the word “individual” to refer to a single T cell sequence read, microbial sequence read, or virus- infected cell. We use “species” to denote a class of individuals, such a T cell clonotype, bacterial operational taxonomic unit (OTU) or viral clone. The term “species richness” denotes the number of species in the population under consideration.

Immunological and microbiological data differ in important respects from ecological data. First, in many immunological and microbiological populations, it may be reasonable to assume that “species” are taxonomically similar, that the spatial distribution of individuals is homogeneous, and that individuals are sampled randomly, independently and with equal probabilities. If made, these simplifying assumptions allow the extrapolation of individual-based rarefaction curves, which depict the expected number of species against the number of individuals sampled [11]–[14]. However, the above assumptions are frequently violated in ecological populations [14]–[18], where unobserved individuals may differ from observed individuals in their colour, physical size, geographical distribution, movement, variety of habitats and relationship to other species [15], and thus remain unobserved despite substantial subsequent sampling. Second, many common assumptions about population structure are inappropriate for immunological and microbiological populations, for example that all species have equal frequencies [19]–[21], or that the functional form of the population distribution is known [22]–[26]. We therefore consider non-parametric estimators.

Non-parametric estimators, such as Chao1 [27], and the abundance-based coverage estimator (ACE) [28], have been proposed. ACE has been suggested to be the best current approach [14], [22], [29] and is widely applied in microbiology and immunology; for example to estimate the diversity of the human gastrointestinal flora [30], human gut metagenome [31], mouse TCR repertoire [32], [33], fungi [34], and the number of HTLV-1 infected cell clones [35]. Although they were originally intended as methods to estimate lower bounds, the Chao1 estimator, and the modified, bias-corrected form Chao1bc [36], have been used to make a point estimate of the number of TCR clonotypes [37], [38], the number of OTUs in hepatitis C virus infection [1], parasite diversity in malaria infection [39] metagenome size [40], the number of integration sites of therapeutic gene therapy vectors [41], soil diversity [42], and again the number of HTLV-1 infected cell clones [35], [43]. In addition to the ACE and the Chao estimator, we also consider two additional non-parametric estimators: the Bootstrap [44] and Good-Turing estimators [45].

Most diversity estimators aim to estimate the species richness in one of two populations of interest: either in the population from which the sample was drawn (e.g. number of microbial species in the gut, given a sample from the gut) or the value where the rarefaction curve saturates (e.g. number of species at the point when further sampling does not yield any new species). These definitions of the population of interest lack flexibility and may be inappropriate or poorly defined for the question in hand. Indeed, if some species are represented by a single individual, the rarefaction curve will not saturate. For many microbiological and immunological questions, an estimator that allows the user to specify the size of the population of interest is desirable. For instance, we may wish to know the T cell repertoire diversity of both the blood and the whole body.

The aim of this study was to identify a suitable method for estimating species richness in immunological and microbiological populations. We tested widely-used estimators on samples of microbiological and immunological populations. We found these estimators performed poorly. We therefore developed and validated a new method to estimate species richness and species frequencies.

We used data from three independent sources: (i) viral populations from human T-lymphotropic virus type-1 (HTLV-1)–infected subjects; (ii) T cell antigen receptor (TCR) clonotype repertoires; and, (iii) infant faecal microbial samples.

HTLV-1 is a retrovirus that mainly infects CD4^+^ T lymphocytes. HTLV-1 spreads within hosts via two routes: *de novo* infection of uninfected cells, and proliferation of infected cells [46]. When an infected cell proliferates, the integrated provirus is replicated with the host genome and a clone of infected cells is generated, each cell carrying a provirus in the same genomic site. Consequently, in each host, HTLV-1 persists in many distinct infected cell clones. We used high-throughput data on the abundance of HTLV-1 infected cell clones in 14 HTLV-1 seropositive subjects [43].

The human gastrointestinal tract contains a densely populated ecosystem of microbes that performs a variety of functions [47]. We obtained high-throughput 16S rRNA sequence data from infant faecal samples. In this study we used observed frequencies of different bacterial operational taxonomic units (OTUs) [48].

T cells are vital to adaptive immunity. The T cell population comprises a diverse repertoire of TCR clonotypes, each defined by the DNA sequence of the expressed TCR. In humans, there are a potential 10^15^–10^20^ different TCR clonotypes [49], but the actual number of clonotypes in one person is estimated to be between 10^6^ and 10^8^ [50]. In this study we used RACE-based data on TCR clonotype abundance. We studied circulating central and effector memory, naïve and total CD4^+^ and CD8^+^ T cells.

## Materials and Methods

### Ethics Statement

Blood samples were donated by HTLV-1^+^ subjects attending the HTLV-1 clinic at the National Centre for Human Retrovirology (Imperial College Healthcare NHS trust) at St. Mary's Hospital, London UK, with fully informed written consent. This study was approved by the UK National Research Ethics Service (NRES reference 09/H0606/106). Parents gave full written informed consent for infant faecal sample collection, and all protocols and procedures were approved by the National Research Ethics Service Committee, U.K. (Southampton and South West Hampshire) (ref: 05/Q1702/119). For the TCR data, leukaphereses were performed on healthy donors who provided written informed consent at the National Institutes of Health, USA. The protocol and use of these samples for immunological investigation were approved by the National Institute of Allergy and Infectious Diseases Institutional Review Board.

### HTLV-1 Data Collection

Previously reported [43] and new high-throughput data on HTLV-1 clonality were analysed. Each HTLV-1 dataset quantifies the abundance of HTLV-1-infected T cell clones. There were 105 datasets, comprising nine samples from each of 11 subjects (three independent samples at each of three time points), and 15 samples from four subjects. All had either HTLV-1-associated myelopathy/tropical spastic paraparesis or were asymptomatic carriers of HTLV-1.

### Microbial Data Collection

The microbial data were derived from faecal samples obtained from 10 infants. DNA was amplified with two sets of PCR primers, generating 20 datasets [48]. Amplicons of the V3-V5 regions of the 16S rRNA gene were generated by PCR using two sets of universal primers. Sequencing data were generated using the Roche 454 GS Junior platform. Analysis was performed using the QIIME pipeline as described previously [48].

### TCR Data Collection

A total of 16 datasets were collected from two subjects, comprising TCR sequences from four phenotypically defined subsets of CD4^+^ and CD8^+^ T-cells: naïve, central memory (CM), effector memory (EM) and total. After flow cytometric sorting and cell lysis, mRNA was extracted and subjected to a non-nested, template-switch anchored RT-PCR using a 3′ TCRB constant region primer as described previously [51]. This approach allows linear and unbiased amplification of all TCRs irrespective of *TRBV* or *TRBJ* gene usage. Paired-end sequencing reactions (each 150 bp) were performed using an Illumina HiSeq 2000 sequencer. Raw FASTQ files were annotated using reference TCRB sequences from the ImMunoGeneTics (IMGT) website (http://www.imgt.org) and a custom-written Java application. Following annotation, the data were filtered to eliminate potential sequencing and PCR errors.

### *Prochlorococcus* Data Collection

*Prochlorococci* are vital to energy and nutrient cycling in the oceanic ecosystem, and the genus contains a highly diverse and abundant population of clades. We analysed publicly available metagenomic data describing clades *Prochlorococcus*. The data were obtained by the Global Ocean Sampling Expedition and contains the frequency of distinct sequence reads of genes of *Prochlorococcus* clades.[52] Sampling sites, sample collection, library construction, fragment recruitment, and determination of *Prochlorococcus* abundances are detailed in [52], [53].

### *DivE* Species Richness Estimator

**Figure 1:**
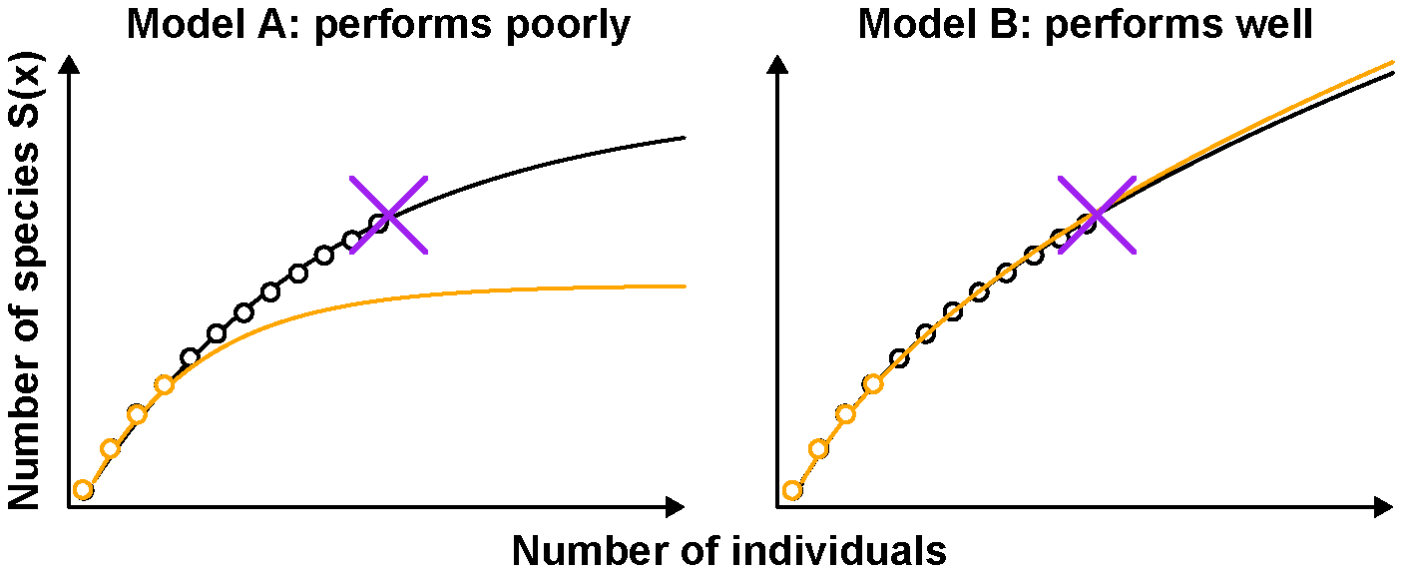
Outline of *DivE* species richness estimator. *DivE* fits many models to rarefaction curves (black) and subsamples thereof (orange). Data is denoted by circles; fits by solid lines. Models are scored according to the following criteria: **i)** ***Discrepancy*** – mean percentage error between data points and model prediction; **ii)** ***Accuracy*** – error between full sample species richness (purple cross) and estimated species richness from subsample; **iii)** ***Similarity*** – area between subsample fit (orange) and full data fit (black); and **iv)** ***Plausibility*** – we require that *S'(x) ≥0* and *S"(x) ≤0*. The best performing models are aggregated and extrapolated to estimate species richness. Model A performs poorly as criteria ii) and iii) are not satisfied. Model B performs well as all criteria are satisfied.

We developed a heuristic approach to estimate species richness, which we named *DivE* (Diversity Estimator) (Figure 1). To calculate the *DivE* estimator, many mathematical models are fitted to multiple nested subsamples of individual-based rarefaction curves. Each model is fitted to all nested subsamples, and is scored on a set of four criteria. The five best-performing models are extrapolated and their respective estimates are aggregated to produce the *DivE* species richness estimate. *DivE* requires an estimate of population size. If the species richness of a wider population is desired, the same models are used but extrapolated to a different population size; this is only justified if the two populations are similar in their spatial distribution of individuals.

The criteria against which each model fit is scored are:

1. ***Discrepancy*** – the mean percentage error between data points and model prediction.
2. ***Accuracy*** – the percentage error between the full sample species richness, and the estimate of full sample species richness from a given subsample.
3. ***Similarity*** – the area between the curve fitted to a subsample and the curve fitted to the full sample, normalized to the area under the curve from the full data, on the interval [0, *N_obs_*], where *N_obs_* is the size of the full data.
4. ***Plausibility*** – the predicted number of species must either increase monotonically or plateau and the predicted rate of species accumulation must either decrease or plateau (i.e. for *S(x)* and *x≥1*, where *x* is the number of individuals, *S′(x) ≥0*, and *S"(x) ≤0*).

The rationale behind each criterion is as follows:

1. ***Discrepancy*** - the model must describe the data to which it was fitted.
2. ***Accuracy*** - from a subsample, the model should predict the full sample species richness.
3. ***Similarity*** - an ideal model will produce identical fits from all subsamples. The smaller the area between the model fits, the better the model.
4. ***Plausibility*** - this criterion requires that, as the observed number of individuals increases, the observed number of species does not decrease and the rate of species-accumulation does not increase; the former is impossible and the latter is implausible (Figure 1).

Criteria 2), 3) and 4) are independent of the fitting process. That is, they are not constraints by which models are fitted; instead they are tests of model performance.

Each model fit is scored on all four criteria. For criteria 1–3, we scored a fit in multiples of empirically chosen precision levels. The precision level for criterion 1 was 0.01%: a score of 1 denotes a model fit where the mean percentage error of the residuals, ε, was less than 0.01%; a score of 2 denotes 0.01%<ε≤0.02% and so on. Criteria 2 and 3 were similarly scored in multiples of 0.5%. Criterion 4 was implemented by giving a score of 500 to model fits that violated either of its conditions; this value was chosen to exceed the score of any model fit that satisfied this criterion.

The final score for each model is an aggregate of the scores of all model fits across subsamples and criteria, and is calculated as follows. First, the score for each criterion is defined as the mean of the scores of all subsample fits for that criterion. The final score for each model is the mean of all criteria scores. The *DivE* species richness estimate is the geometric mean of the estimates provided by the five best-performing (i.e. lowest-scoring) models.

A list of 58 candidate models (Text S1) was chosen from an online repository [54]. Many of these (e.g. logistic, logarithmic, hyperbolic) are widely used in population ecology [11], [55]. Models were fitted by least squares regression using R version 2.14.2 [56] with the package FME [57]. Global fitting was performed using Price's algorithm [58] followed by local fitting using the Levenberg-Marquardt algorithm [59].

### Study Design

We evaluated *DivE* and five non-parametric estimators: the Chao1 bias-corrected estimator (Chao1bc) [36], the abundance-based coverage estimator (ACE) [28], the Bootstrap estimator [44], the Good-Turing estimator [45], [60], [61] and the widely-used negative exponential model [11], [12], [36], [62], [63]. ACE and Chao1 [27], have been suggested as best practice [12], [14], [22], [29], [64] and are widely applied in microbiology and immunology [1], [30], [32], [34], [35], [37], [39]–[43]. For ACE, “abundant” species were defined as those with an observed frequency of greater than 10, as recommended in [64].

Due to differences between estimators and between datasets, we conducted multiple, distinct evaluations and validations. We first evaluated, for each estimator, the relationship between estimated diversity and sample size, using the estimates produced from a series of successively smaller, randomly generated *in silico* subsamples of observed data. For the microbial and TCR data respectively, five and six equidistant subsample sizes were chosen from each observed dataset. For the HTLV-1 data, subsample sizes were chosen to be approximately equidistant; however some were removed due to runtime constraints. See Table S1 for further details. Second, we measured the accuracy of *DivE* by comparing the estimated species richness *Ŝ_obs_* at the size of the full dataset *N_obs_* from each subsample to the (known) species richness *S_obs_* in the full data. Using the same method, we compared *DivE* to the second order bias-corrected Akaike Information Criterion (AIC_c_) [65], [66]. Third, the TCR data have rarefaction curves which plateau. Using smaller subsamples of this data and making the assumption that the species richness of the full data is equal to that of the entire population, we were able to evaluate the accuracies of all estimators together. Finally for 11 of the 14 HTLV-1 patients detailed in Table S1, three samples were taken at a single time point. For each time point, the three samples were pooled and used as a practical test of *DivE*'s ability to predict species richness in larger samples.

### *DivE* Frequency Distribution Generation Algorithm

**Figure 2:**
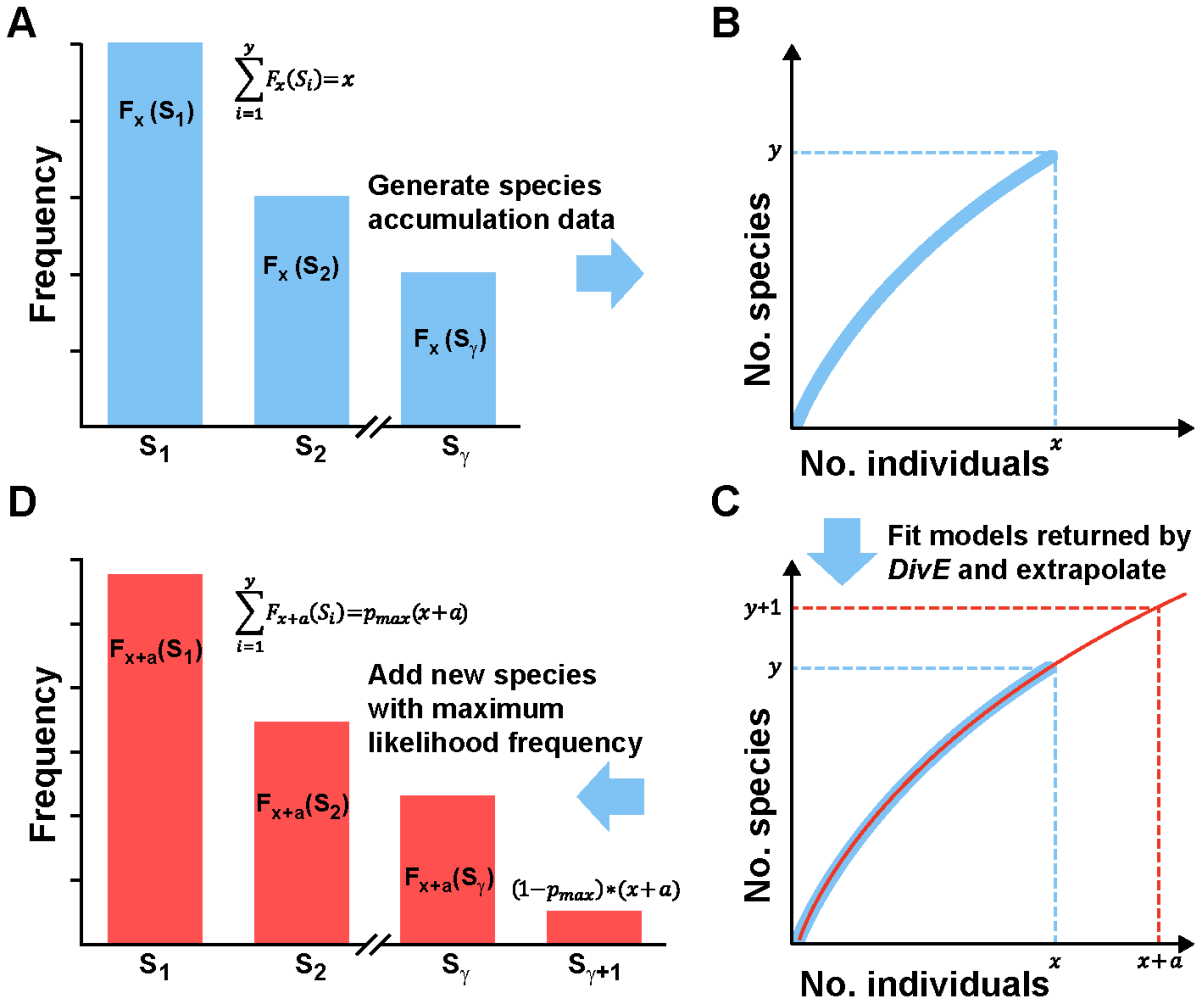
Outline of *DivE* distribution generation algorithm. **A** Truncated species frequency distribution with *x* individuals distributed among *y* species. The frequency of species *S_i_* after sampling *x* individuals is denoted *F_x_(S_i_)*. **B** Species accumulation data generated from frequency distribution. **C** An aggregate of the best performing models as returned by *DivE* is used to extrapolate to point *(x+a, y+1)*, where the next species is predicted. **D** Species *S_y+1_* is assigned a frequency of *(1 - p_max_)(x+a)*, where *p_max_* is the maximum-likelihood proportion of individuals occupied by the *y* previously observed species. The remaining *p_max_(x+a)* individuals are distributed among species *S_1_*, …, *S_y_* in proportion to their observed relative frequencies at *x*. Steps **C** and **D** are repeated until the predicted species richness is reached. See Text S1 for further details.

In addition to species richness, we wanted to estimate the population frequency distribution. Because of the considerable structural variation between and within immunological and microbiological populations, we developed a general method which does not assume the analytical form of the population structure. This algorithm uses the *DivE* estimator combined with observed abundances (Figure 2). See Text S1 for details. The algorithm was applied to multiple random subsamples of observed data. The estimated distributions were then compared to the full data frequency distribution using two measurements: (i) error, defined as the sum of discrepancies in species frequencies between estimated and observed (full) distributions, divided by the number of individuals in the observed distribution, i.e. error  = 
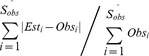
and (ii) percentage error between the Gini coefficients of the estimated and observed distributions. The Gini coefficient is an index of dispersion used widely in epidemiology, sociology, biology, and ecology [43], [67].

## Results

### Comparison of Estimators: Relationship between Sample Size and Estimated Diversity

Each species richness estimator (Chao1bc [36], Bootstrap [44], ACE [28], Good-Turing [45], the negative-exponential model [12] and *DivE*) was applied to random subsamples of observed data. We used linear regression to calculate the average proportional increase in estimated diversity as a function of the proportional increase in sample size. Sample size and diversity were normalized respectively to the smallest sample and the estimated diversity at the smallest sample. For example, a “normalized gradient” of 0.5 would mean that, on average, an increase of 10% in sample size would produce a 5% increase in estimated diversity. A value of zero would signify no bias with sample size.

**Figure 3:**
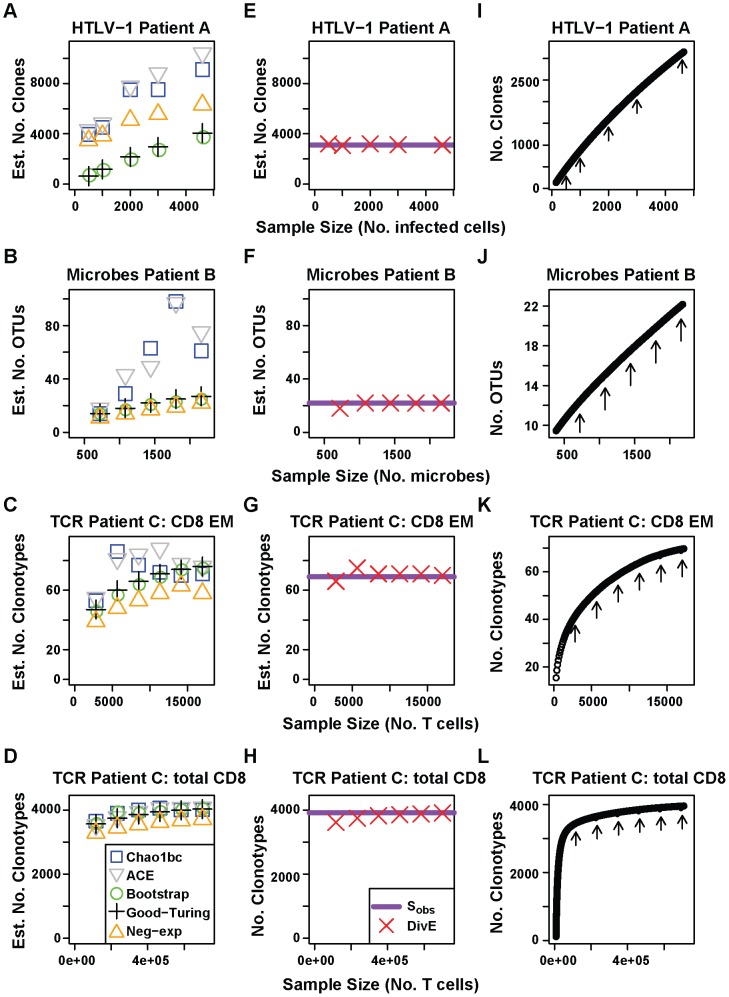
Comparison of species richness estimators. **A–D** The Chao1bc (blue), ACE (grey), Bootstrap (green), Good-Turing (black), and negative-exponential estimators (orange) are applied to *in silico* random subsamples of observed data. Examples for HTLV-1, microbial, and TCR data are shown. Estimates systematically increase with sample size in datasets where rarefaction curves do not plateau (e.g. in **I**, **J**, **K**). Where rarefaction curves do plateau (e.g. in **L**), estimates are consistent. **E–H** *DivE* (red) is applied to same subsamples as the other estimators. Performance of *DivE* was evaluated by comparing the error of estimates (*Ŝ_obs_*), to the (known) number of species *S_obs_* in the full observed data (purple line), i.e. error  = |*S_obs_* - *Ŝ_obs_*| /*S_obs_*. In all datasets, *DivE* accurately estimates the species richness of the full observed data from subsamples of that data. **I–L** Corresponding HTLV-1, microbial and TCR rarefaction curves: arrows denote the size of the subsample to which each estimator was applied.

**Figure 4:**
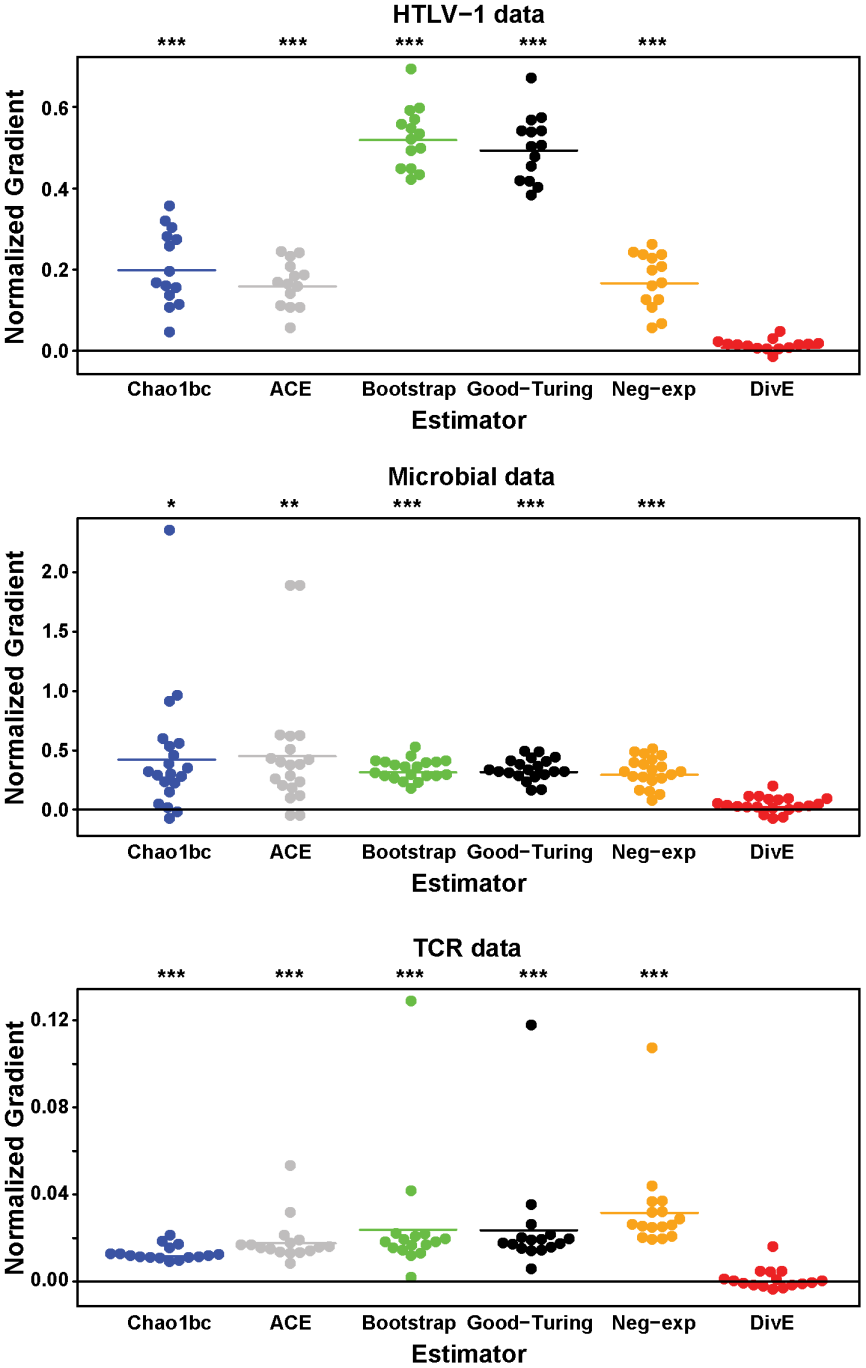
Comparison of estimators: Effect of sample size on estimated diversity. Normalized gradients measuring proportional increase in estimated diversity against proportional increase in sample size. Normalized gradients (shown for each estimator and each patient data set in Table S1) were calculated by linear regression. For the HTLV-1 and microbial data, all estimators except *DivE* show large normalized gradients that are significantly positive. The TCR normalized gradients, though significantly positive, are small and do not show a substantial bias with sample size. *, **, and *** signify p<0.01, p<0.001, and p<0.0001 respectively; two-tailed binomial test (n = 14, 16, 20 for the HTLV-1, TCR and microbial data respectively).

The existing estimators performed poorly when applied to the HTLV-1 and microbial data: estimates systematically increased with sample size. In contrast, *DivE* produced consistent estimates that showed no obvious relationship with sample size (Figures 3 and 4). Across subjects and for all methods except *DivE*, estimates showed significant positive normalized gradients (p<0.01 for every estimator, n = 14; two-tailed binomial test) ranging between 0.17 and 0.52 for the HTLV-1 data and 0.3 to 0.45 for the microbial data (Figure 4). Conversely, the normalized gradients produced by *DivE* did not differ significantly from zero (p = 0.18, n = 14; two-tailed binomial test), and were much smaller (0.0081 and 0.022 for the HTLV-1 and microbial data respectively) (Figure 4). In any specified population there is only one value of species richness, and an accurate estimator will arrive at this value regardless of sample size. An increase in estimate magnitude with sample size implies that estimates of a population's species richness would increase if e.g. greater blood volumes were drawn or technique sensitivity was improved.

The existing estimators were less biased when applied to the TCR data, and estimates were largely consistent. Although the normalized gradients were still significantly positive (p<0.0001 for each estimator except *DivE*, n = 16), their magnitudes were substantially lower than for the HTLV-1 and microbial data. However, existing estimators again increased with sample size for the effector memory (EM) CD8^+^ T cell population from the same subject. These observations can be explained with reference to the TCR rarefaction curves (Figure 3). With the exception of the CD8^+^ EM dataset (for which the subsample sizes were considerably smaller), each TCR rarefaction curve reached a plateau, implying that the vast majority of observed clonotypes were encountered early. In contrast, the CD8^+^ EM rarefaction curve did not plateau, suggesting that further sampling would reveal more CD8^+^ EM clonotypes. In common with the microbial and the HTLV-1 datasets, *DivE* performed well for all TCR datasets, producing consistent results from all subsample sizes. To make sure that the smallest subsamples did not disproportionately contribute to the observed gradients, we repeated the above analysis using only estimates from the largest three subsamples in each patient dataset, which showed almost identical results (Figure S1).

### Comparison of *DivE* and Second Order Bias-Corrected Akaike Information Criterion (*AIC_c_*)

The best-performing models were largely consistent within patients and between subsamples for the microbial and TCR data, although less so for the HTLV-1 data. Ideally, model selection would be consistent across all subsamples. Deviation from this will result in a discrepancy between *S_obs_* and *Ŝ_obs_*. This discrepancy is quantified in Figure 3 (middle column) and in Table S2. To ensure the four criteria provide a useful metric of model performance, we compared *DivE* to the second order bias-corrected Akaike Information Criterion(AIC_c_) [65], [66]. DivE's mean errors (between the species richness of the full data Sobs and *Ŝ_obs_*) were 3.3%, 1.0%, and 4.0% for the HTLV-1, TCR and microbial data respectively. These were lower than the corresponding errors of 6.7%, 1.1%, and 7.5%, produced when models were scored by the AICc. This effect was more marked when we considered estimates from small subsamples, defined as those comprising at most 50% of the observed data (Table S2). However, the differences between errors were smaller for the TCR data, perhaps also due to the saturating rarefaction curves in these samples.

### Comparison of Estimators: Accuracy of Diversity Estimate

When rarefaction curves reach a plateau, we can assume that the value of the plateau is approximately equal to the species richness of the entire population, which the existing estimators aim to estimate. Thus it is appropriate to evaluate *DivE* and the existing estimators together using TCR rarefaction curves which plateau. We took random subsamples of 0.5%, 1%, 2%, 5%, and 10% of the total CD4^+^ and CD8^+^ cells for subjects C and E. We then applied each estimator to each subsample and measured its error ( = |*S_obs_* - *Ŝ_obs_*| /*S_obs_*) (Table 1, Figure S2). *DivE*'s median error was 6.7%, substantially lower than respective median errors of 43.8%, 42.8%, 65.3%, 61.7%, and 50.7% for the Chao1bc, ACE, Bootstrap, Good-Turing and negative exponential estimators (p<0.0005 for each estimator comparison with *DivE*, n = 20; two-tailed binomial test)

**Table 1:** Comparison of estimator performance for TCR data.

Estimator	Median Error* (%)	P-value†
Chao1bc	43.8	0.0004
ACE	42.8	0.0004
Bootstrap	65.3	<0.0001
Negative-exponential	50.7	<0.0001
Good-Turing	61.7	<0.0001
*DivE*	6.7	NA

*Median absolute percentage error between *S_obs_* and *Ŝ_obs_*.

† p-value of the significance of the differences between the errors of each estimator and *DivE* (n = 24; two-tailed binomial test).

**Figure 5:**
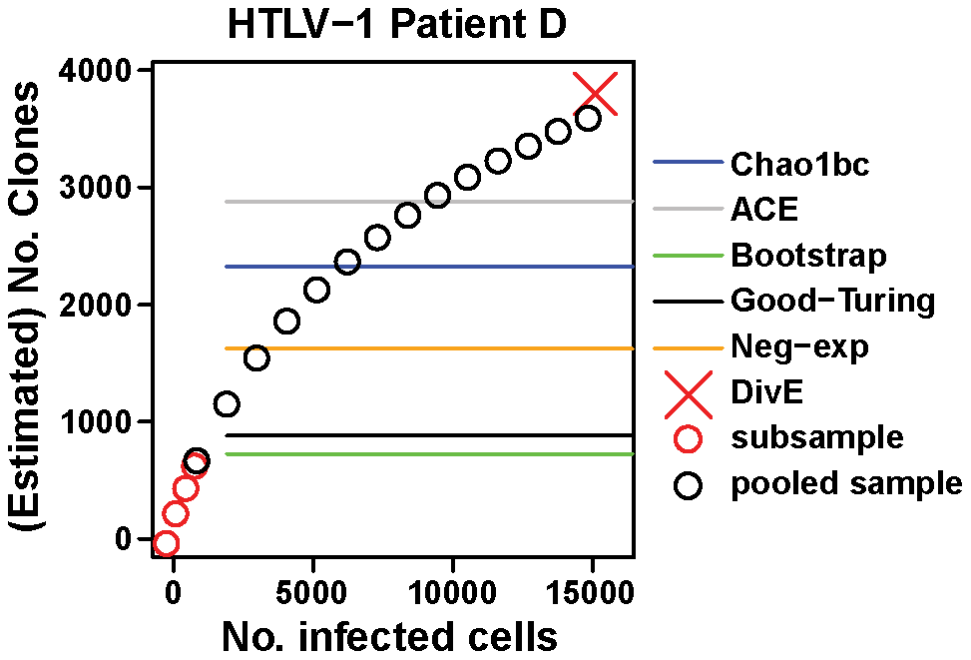
Existing estimators underestimate diversity in HTLV-1 infection. For HTLV-1 Patient D, three samples are pooled. Rarefaction curves from the pooled sample (black circles) and a subsample (red circles) are shown. Chao1bc, ACE, Bootstrap, Good-Turing and negative exponential estimates (blue, grey, green, black, and orange lines respectively) from the subsample, and *DivE* estimates (red cross) from the same subsample are plotted. Existing estimators produce a single estimate of diversity, and so their estimates are shown as lines. The diversity in the blood must be at least as great as that observed by pooling the samples. All existing estimators estimate the total diversity to be less than that observed. Given that the observed diversity is likely to be a small fraction of the total diversity this represents a considerable error. We used *DivE* to produce two estimates: the diversity in the pooled sample (i.e. in 15000 cells, red cross) and the total diversity of the blood. *DivE* accurately estimates the pooled sample species richness from the subsample, but also predicts higher values of species richness in the blood, consistent with the unseen clones implied by the pooled rarefaction curve. See Figure S3 for further examples.

As neither the HTLV-1 nor the microbial data exhibit rarefaction curves that plateau, we cannot apply the same analysis to these datasets. Instead we took advantage of the fact that, for 11 of the 14 HTLV-1 subjects, the data comprised three time points,with three samples drawn at each time point in immediate succession from the subject. For a given subject and a single time point, the three samples were combined *in silico* to produce a single pooled sample. We compared the observed species richness of the pooled sample to each estimator's estimates from a subsample (Figure 5, Figure S3). The total blood diversity must be at least as great as that observed by pooling the samples. However, all existing estimators estimate the total diversity to be less than that observed. Based on a single subsample, the Chao1bc, ACE, Bootstrap, Good-Turing and negative exponential estimators respectively estimate medians of 27.0%, 12.7%, 71.1%, 65.5%, and 47.6% fewer clones than observed in the pooled samples (n = 11). Since the pooled samples do not saturate, and since the blood contains approximately 10^5^ times more infected cells than the pooled sample, the diversity observed in the pooled sample is likely to be a small fraction of the total diversity. Since the existing estimators produce estimates lower than the pooled sample diversity, let alone total blood diversity, this represents a considerable error. We used *DivE* to produce two estimates: the pooled sample diversity and blood diversity. From the subsamples *DivE* estimated a median of 2.6×10^3^ clones in the pooled samples, a median error of 2.5% (n = 11) (Figure 5, Figure S3). Additionally, *DivE* estimated 2.8×10^4^ clones in the blood, approximately one log higher than the observed pooled sample diversity. Whilst we cannot determine whether or not this is accurate it is at least plausible, considering that it is not less than the diversity of the pooled sample, that the sampling fraction is very small, and that the rarefaction curve has not reached a plateau.

### Estimate Error as a Function of Data Curvature

Next we sought to identify conditions under which *DivE* would be prone to error and should not be applied. When the observed rarefaction curve is linear, the data imply a constant rate of species accumulation, and so provide little information on how quickly the rate of species accumulation will decrease. This is usually indicative of severe under-sampling. We predicted that *DivE* will fail to give accurate estimates given such a near linear rarefaction curve. We tested this prediction by calculating the error in the *DivE* estimates as a function of rarefaction data curvature.

The curvature *C_p_* was quantified by the area between the observed rarefaction curve and a linear rarefaction curve, as a fraction of the maximum possible area, which occurs when the rarefaction curve saturates immediately. *C_p_* can take values between 0 and 1, where 1 reflects perfect saturation and 0 reflects a constant rate of species accumulation (Figure S4). We took additional samples of 0.1% of the total CD4^+^ and CD8^+^ cells for subjects C and E to obtain lower curvature values.

**Figure 6:**
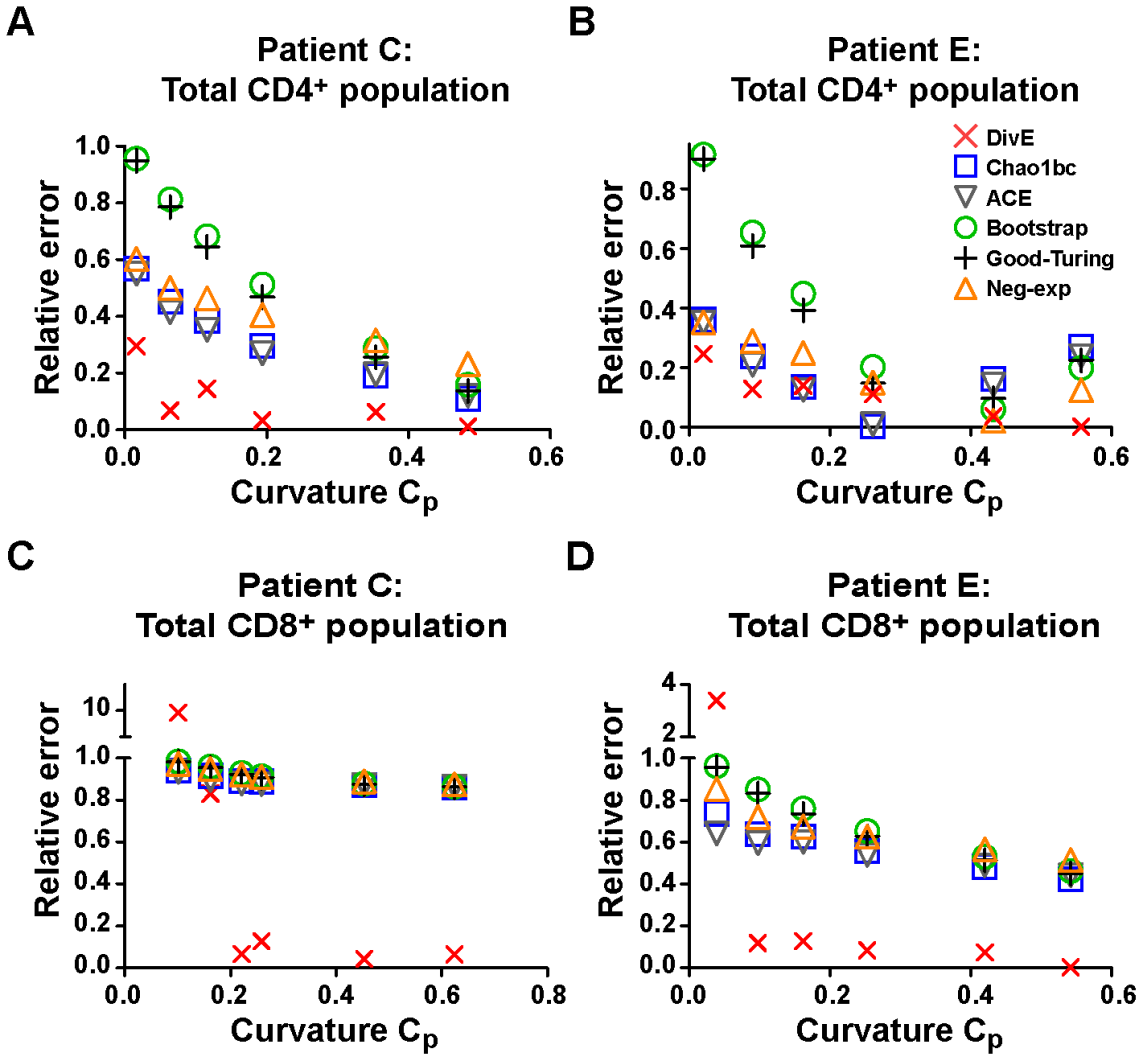
Test of species richness estimators at different values of curvature parameter (*C_p_*) using TCR data. The curvature parameter *C_p_* is plotted against the relative error (|*S_obs_* - *Ŝ_obs_*| /*S_obs_*) of each estimator. Four patient data sets are shown: **A** total CD4^+^ from patient C; **B** total CD4^+^ from patient E; **C** total CD8^+^ from patient C; **D** total CD8^+^ from patient E. Each point represents an estimate from a subsample of data. Note the plots have different y-axis scales and the y-axes in **C** and **D** are segmented. Broadly, the accuracy of all estimators improves as *C_p_* increases, and this increase is more pronounced for *DivE*. From *C_p_*>0.1, *DivE* generally outperforms the existing estimators, but is prone to error at very low values of *C_p_*., when the rarefaction curve implies a near-constant rate of species accumulation.

As expected, at very low curvatures (0.016≤*C_p_*≤0.101), *DivE* was prone to overestimation and performed poorly (Figure 6), with median error 0.23. However, for under-sampled populations of intermediate curvature (0.11≤*C_p_*≤0.62) *DivE* improved markedly (median error  = 0.06), and typically outperformed the other estimators (Figure 6, Table S3). Finally, all estimators perform well when the curvature is high and most of the diversity has been observed (Figure 3D, 3H and 3L).

We next tested *DivE* using the *Prochlorococcus* data [52], with multiple subsamples of increasing curvature (as for the TCR data). At low curvatures *DivE* again performed poorly, but it became more accurate as the curvature increased. For under-sampled populations of intermediate curvature, *DivE* again outperformed the other estimators, although the differences between the estimator errors were not as dramatic as with the TCR data (Figure S5).

Very low curvatures suggest severe under-sampling and researchers should exercise caution with such data. It is unlikely that any species richness estimator will be accurate or informative in such cases.

### Example Application: Estimated Number of HTLV-1 Infected Cell Clones

In both HTLV-1 infection and infection with the related bovine leukaemia virus (BLV), accurate determination of the number of infected cell clones in the host is critical to understanding retroviral dynamics and pathogenesis [68]-[71]. Here we make two different estimates of the number of HTLV-1 infected cell clones: (i) in the circulation; and, (ii) in the whole body. See Text S1 for details of HTLV-1 population size estimation.

The mean estimated number of clones in the circulation in a single host was 2.9×10^4^. It is unknown whether the population structure of HTLV-1 clones in the blood reflects that in solid lymphoid tissue and the spleen. If we assume that these two populations have similar structures, and thus that it is justified to extrapolate to the whole body, we obtain an average of 6.2×10^4^ clones, i.e. approximately only twice as many clones, although there are >300 times as many infected cells in the body as the blood. These new estimates in the blood and body are approximately 1 and 1.3 logs higher respectively than those calculated using ACE and Chao1bc (p<0.0001, two-tailed paired Mann-Whitney U-test), and >2 logs higher than previously published estimates (Figure S6) [35], [43], [69], [72].

### *DivE* Uncertainty

Because of its heuristic nature, *DivE* lacks formal statistical confidence intervals. Uncertainty in the estimates produced by *DivE* has two sources: parameter values in each respective model (within-model variation), and the choice of model (between-model variation). Using standard errors of parameter estimates to calculate confidence intervals ignores uncertainty from model selection. Information theoretic approaches that take account of model selection uncertainty have become increasingly common in ecology [73], [74] and elsewhere. There are broadly two approaches: i) computing AIC weights, and ii) repeated resampling and model ranking to determine bootstrap model selection probabilities [66]. However, neither approach is appropriate in our case. We do not rank models using AIC since this produces less accurate estimates than *DivE* (Table S2), and so we cannot use AIC weights to derive confidence intervals. Further, since there is a systematic bias towards lower species richness in bootstrap samples (Figure S7), a similar bias may be introduced in the estimation of bootstrap model selection probabilities, leading in turn to a bias in species richness estimation. Systematic underestimation in bootstrap samples is particular to species richness estimation: this does not highlight a general problem with resampling to quantify model selection uncertainty. As a pragmatic indicator of estimate variability, we use the range of estimates produced by the five best-performing models; the geometric mean of these five models is taken as the point estimate (Table S4).

### Distribution Generation Algorithm

**Figure 7:**
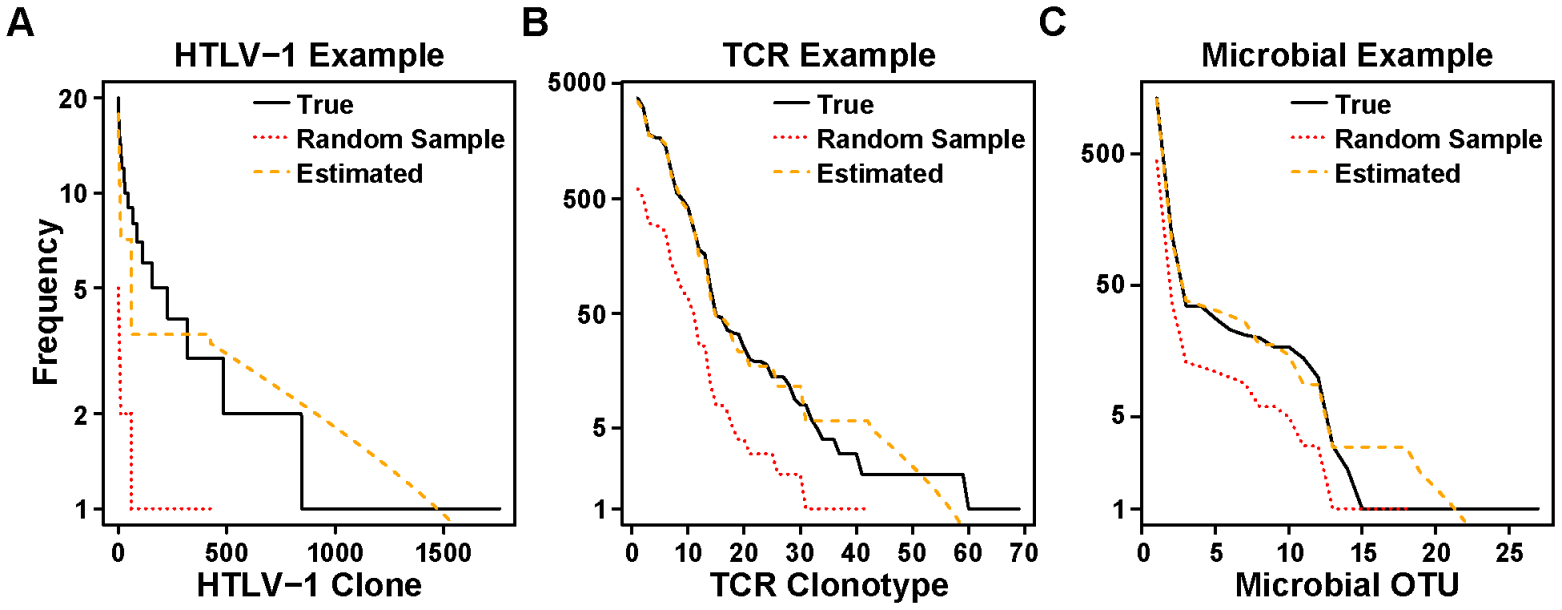
Validation of *DivE* distribution generation algorithm. The *DivE* distribution generation algorithm (Figure 2) was applied to random samples (red dashed) of observed data (black solid). Accuracy was evaluated by comparing the estimated distribution (orange dashed) to the true distribution of the full observed data (black). Examples for HTLV-1 **A**, TCR **B** and microbial datasets **C** are shown.

The distribution generation algorithm was reasonably accurate for the HTLV-1 data, and considerably more accurate for the TCR and microbial data. The mean error between the estimated and true distributions was 32.1%, 2.9%, and 4.9% for the HTLV-1, TCR and microbial data respectively. The mean error between the estimated and true Gini coefficients was 7.5%, 0.9%, and 2.2% for the HTLV-1, TCR and microbial data respectively (Table 2). For the HTLV-1 data, the algorithm underestimated the abundance of the largest clones, but we did not observe this effect in the TCR and microbial data (Figure 7).

**Table 2:** Performance of *DivE* frequency distribution generation algorithm.

Data Source	Mean Error (%)*	Mean Gini Error (%)†
HTLV-1	32.1	7.5
TCR	3.7	1.2
Microbial	6.0	1.1

*Mean error across all subjects and all small subsamples, for each data source. Small subsamples were defined as those ≤50% of the size of the observed each patient data set. Error defined as the sum of absolute discrepancies between true and estimated frequency distributions, divided by area under true distribution.

† Mean percentage error across all subjects and all small subsamples in the Gini coefficients of the true and estimated distributions.

## Discussion

We wished to estimate species richness in three microbiological and immunological datasets. Initially we used estimators that are reported to perform well in ecology [12], [34], [36], [60], [61], [75]. In the datasets with rarefaction curves that did not plateau, these estimators were biased by sample size. For datasets with rarefaction curves that did plateau, estimates were consistent, but in such cases estimators contribute little information because approximate species richness is already known. Comparable results have been reported elsewhere [12], [16], [62]. By combining data from multiple independent HTLV-1 samples, we showed that these estimators substantially underestimated species richness.

We then developed a new approach, *DivE*, to estimate species richness and frequency distribution. In our first validation, *DivE* consistently and accurately estimated the diversity of the observed data from incomplete subsamples of that data. We subsequently determined conditions where *DivE* would fail and should not be applied. When the rarefaction curvature was low and the data implied a near-constant species-accumulation rate, *DivE* was prone to overestimation. However, in under-sampled populations of intermediate curvature, *DivE* substantially improved. The *DivE* distribution generation algorithm performed with reasonable accuracy (Table 2, Figure 7).

We argue that biologically meaningful and useful estimators should be able to estimate species richness in a specified population. This is not the case with the existing estimators we tested. In contrast, *DivE* can estimate diversity in any given population size. However, population size estimation can be nontrivial [76]–[78]. In spatially homogeneous populations with equiprobable detection of individuals, estimating population size through scaling by area or volume is justifiable e.g. scaling from cells in 50 ml of blood to cells in the total blood volume. When population size estimates are unavailable, it is still usually possible to provide meaningful diversity estimates, e.g. the number of microbes per gram of faeces. *DivE* may also be useful in deciding the depth of sampling required for an adequate census. Deeper sampling may require more DNA sequencing or a larger tissue sample from a patient, and so minimizing sampling depth has financial and ethical benefits. This is not possible with the other estimators we tested.

The HTLV-1 data consisted of absolute species counts, and so we could estimate HTLV-1 diversity. Microbial and TCR datasets were used only for validation as these data consisted of sequence reads and not absolute counts. To the extent that read abundances differ from absolute counts, such data cannot be used to estimate species richness with any abundance-based estimator (e.g. *DivE*, Chao1bc, and ACE). Over-amplification by PCR may generate a saturating rarefaction curve that is not due to sampling depth, falsely implying that the majority of species have been observed. This can be seen in our TCR data: plateaus were far lower than previously reported diversity estimates [50], [79]. However, absolute counts can often be obtained (e.g. by spiking a sample with a known quantity of identifiable individuals or by barcoding to identify PCR duplicates).

It is unlikely that sequencing error influenced our HTLV-1 diversity estimates, because sequencing error cannot systematically alter proviral integration site mapping. However, species richness estimates from TCR or microbial data are likely to be susceptible to sequencing error. Sequencing error can falsely increase diversity, and this will influence species richness estimates using any estimator; researchers must therefore exercise caution when analysing such data; ideally by preprocessing the data to remove error prior to further analysis. Caution must also be exercised when assuming that the spatial distribution of individuals is uniform. We believe that these assumptions are reasonable for the blood, but skin tissue for example may be more clustered.

*DivE* is conceptually simple but can be computationally intensive to implement. When applying *DivE* to a new type of data it is necessary to ascertain which models perform best. This requires that many models be fitted to multiple subsamples. If, for a particular data type, a given set of models performs consistently well, application becomes much quicker because only these models need to be fitted, and it is no longer necessary to fit all models to all subsamples. In our analysis we found that five models performed consistently well, and so we have used the aggregate of the five best-performing models in our estimates. Since the optimal number of models may differ between datasets, we advocate careful analysis of model scores to decide how many models should be aggregated. The *DivE* estimator has been provided as an R package, available at http://cran.r-project.org/web/packages/DivE/index.html.

In summary, we have developed and validated a new approach to estimate species richness and distribution that significantly outperformed existing estimators of biodiversity in the datasets we examined.

## Supporting Information

Figure S1 **Estimator bias with sample size not due to subsamples.** As for Figure 4, except that normalized gradients calculated using only largest three subsamples. For the HTLV-1 and microbial data, all estimators except *DivE* again show large normalized gradients that are significantly positive. The TCR normalized gradients, show no bias with sample size. *, **, and *** signify p<0.05, p<0.01, and p<0.001 respectively; two-tailed binomial test (n = 14, 16, 20 for the HTLV-1, TCR and microbial data respectively).
(TIF)

Figure S2 **Comparison of estimators: Accuracy of diversity estimates using TCR data.** Random subsamples of 0.5%, 1%, 2%, 5%, and 10% of the total CD4^+^ and CD8^+^ cells for subjects C and E were taken, and each estimator was applied to each subsample. These populations have rarefaction curves that plateau, so making the assumption that the value of the plateau *S_obs_* is the diversity of the whole population, the distribution of errors for each estimator ( = |*S_obs_* - *Ŝ_obs_*| /*S_obs_*) is shown.
(TIF)

Figure S3 **Existing estimators underestimate diversity in HTLV-1 infection.** As for Figure 5. For each patient, three independent samples are pooled. Rarefaction curves from the pooled sample (black circles) and a subsample (red circles) are shown. Chao1bc, ACE, Bootstrap, Good-Turing and negative exponential estimates (blue, grey, green, black, and orange lines respectively) from the subsample, and *DivE* estimates (red cross) from the same subsample are plotted. All estimators except *DivE* typically estimate fewer clones than observed in pooled sample. In contrast, *DivE* accurately estimates the pooled sample species richness from the subsample.
(TIF)

Figure S4 **Rarefaction curvature parameter** ***C_p_*** **.** Rarefaction curves (dashed) and lines of constant rate of species-accumulation and perfect saturation (solid) are shown. Areas between the line of constant rate of species-accumulation and the rarefaction curve (***A***), and between the rarefaction curve and the line of perfect saturation (***B***) are indicated. Note *C_p_* = 0 when the rarefaction curve is linear.
(TIF)

Figure S5 **Performance of species richness estimators in metagenomic data.** The curvature parameter *C_p_* is plotted against the relative error (|*S_obs_* - *Ŝ_obs_*| /*S_obs_*) of each estimator. Each point represents an estimate from a sample from the *Prochlorococcus* data. As with the TCR data, *DivE* typically outperforms the other estimators from *C_p_*≈0.1 onwards. As predicted, *DivE* is prone to error at lower values of *C_p_*, but becomes more accurate as *C_p_* increases.
(TIF)

Figure S6 **Diversity estimates in HTLV-1 infection by estimator.** Each estimator was applied to 105 patient datasets, from 14 different HTLV-1^+^ subjects. All subjects either had HTLV-1-associated myelopathy/tropical spastic paraparesis or were asymptomatic.
(TIF)

Figure S7 **Rarefaction plots from bootstrap samples of HTLV-1, TCR, and microbial data.** Rarefaction plots from 100 bootstrap samples (grey) for each of **A** HTLV-1, **B** TCR, and **C** microbial data. The species richness of the bootstrap samples is at most the species richness of the original data (black), and is substantially less in the majority of cases, although this effect is less noticeable with the TCR data.
(TIF)

Table S1 **Subsamples used in analysis of relationship between sample size and estimated diversity, and in comparison of** ***DivE*** **with AIC_c_.** 1 Where there were multiple samples at multiple time points in a given HTLV-1-infected subject, a single sample at a single time point was chosen at random.
(PDF)

Table S2 **Comparison of estimates produced by** ***DivE*** **and by weighted, second order Akaike's Information Criterion (** ***AIC_c_*** **).** 1 Average percentage error between *S_obs_* and *Ŝ_obs_* for small subsamples for each data source. Small subsamples were defined as those ≤50% of the size of each patient data set. 2 Large subsamples defined as those >50% of the size of each patient data set. 3 Average percentage error between *S_obs_* and *Ŝ_obs_* across all patient datasets and subsamples for each data source error.
(PDF)

Table S3 **Estimator error variation with curvature in TCR data.** * Median absolute percentage error between *Sobs* and *Ŝobs*. † Low curvatures *Cp* in range 0.016≤*Cp*≤0.101, intermediate curvatures in range 0.11≤*Cp*≤0.62. ‡ p-value of the significance of the differences between the errors of *DivE* and each other estimator, for each curvature range.
(PDF)

Table S4 ***DivE*** **species richness estimates for HTLV-1 data.**
(PDF)

Text S1 **Additional supporting information.**
(PDF)
