# Supplementary figures and images for: Quantification of HTLV-1 Clonality and TCR Diversity
Source: PLoS Comput Biol. 2014 Jun 19;10(6):e1003646. doi: 10.1371/journal.pcbi.1003646 (PMC4063693; doi:10.1371/journal.pcbi.1003646)

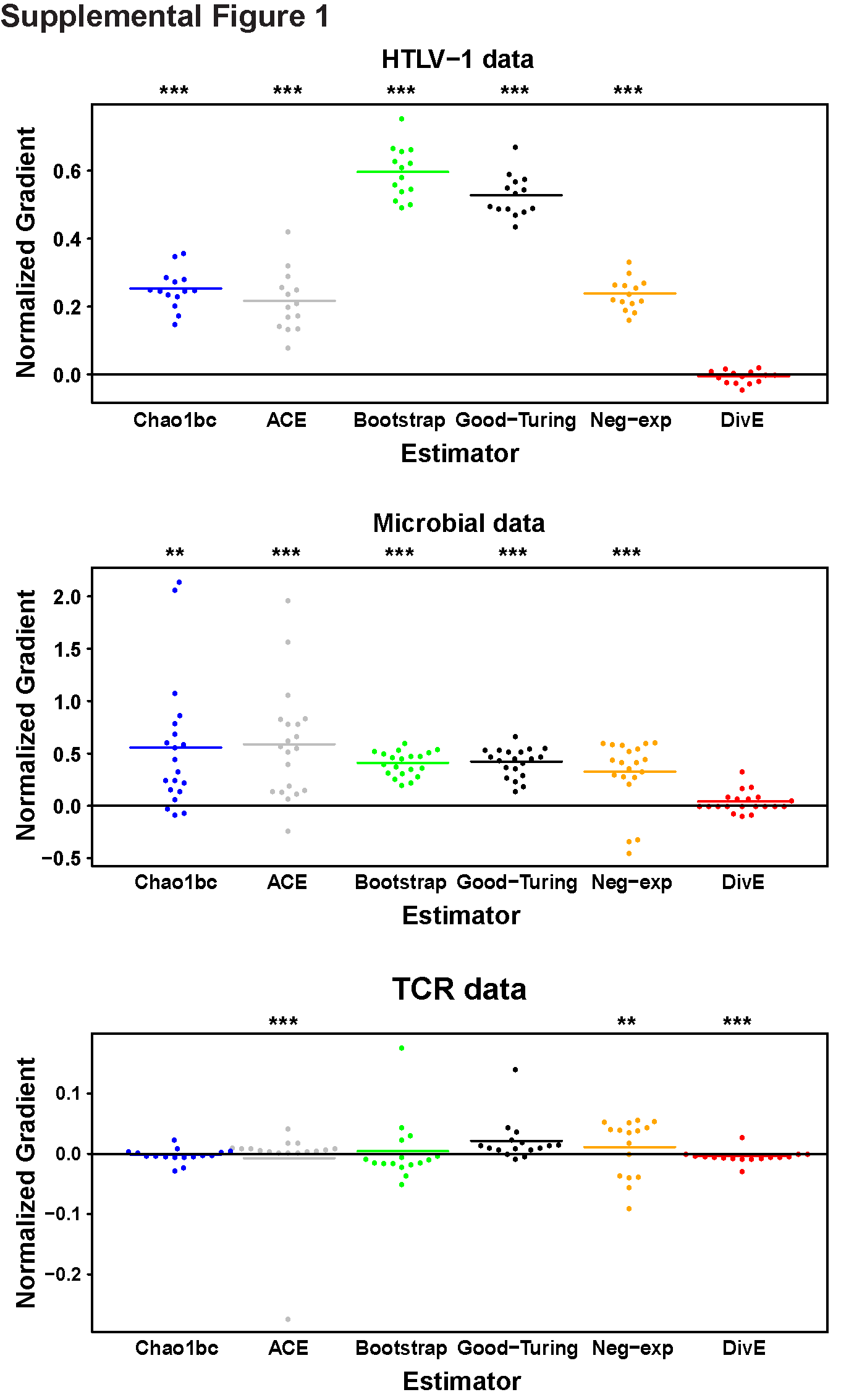

Supplement: Figure S1 — Estimator bias with sample size not due to subsamples. As for Figure 4, except that normalized gradients calculated using only largest three subsamples. For the HTLV-1 and microbial data, all estimators except DivE again show large normalized gradients that are significantly positive. The TCR normalized gradients, show no bias with sample size. *, **, and *** signify p<0.05, p<0.01, and p<0.001 respectively; two-tailed binomial test (n = 14, 16, 20 for the HTLV-1, TCR and microbial data respectively). (TIF) [file pcbi.1003646.s001.tif]

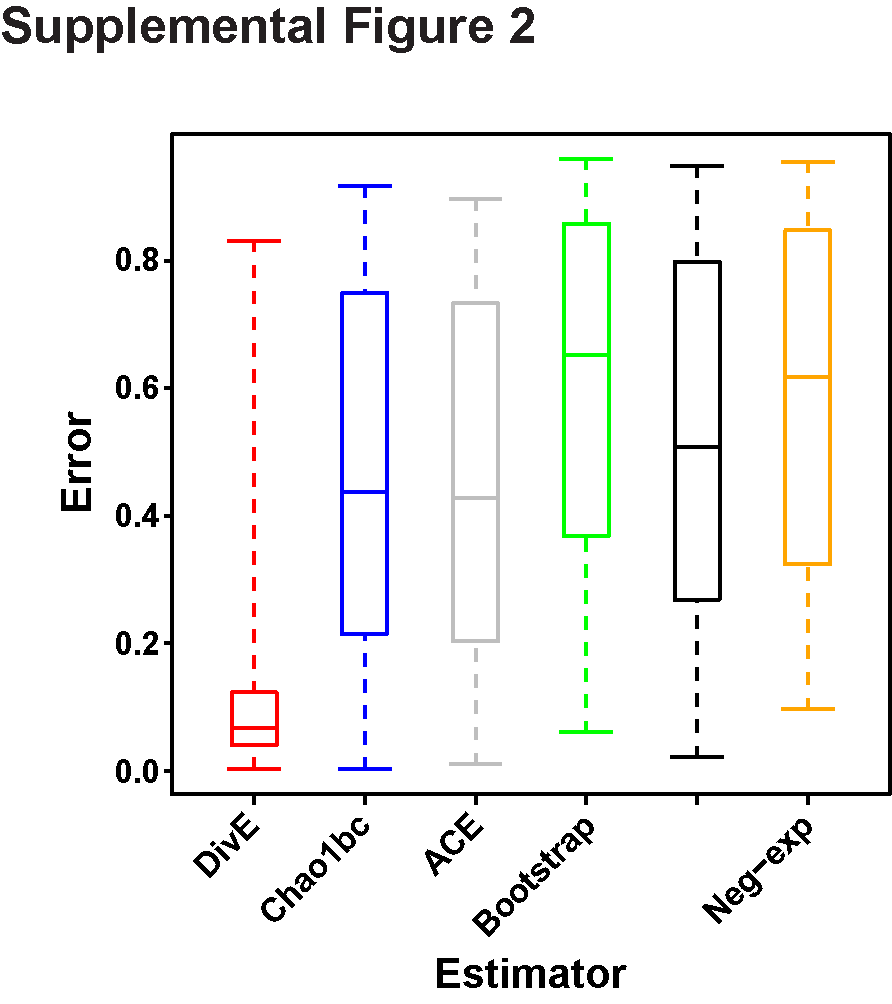

Supplement: Figure S2 — Comparison of estimators: Accuracy of diversity estimates using TCR data. Random subsamples of 0.5%, 1%, 2%, 5%, and 10% of the total CD4+ and CD8+ cells for subjects C and E were taken, and each estimator was applied to each subsample. These populations have rarefaction curves that plateau, so making the assumption that the value of the plateau Sobs is the diversity of the whole population, the distribution of errors for each estimator ( = |Sobs - Ŝobs| /Sobs) is shown. (TIF) [file pcbi.1003646.s002.tif]

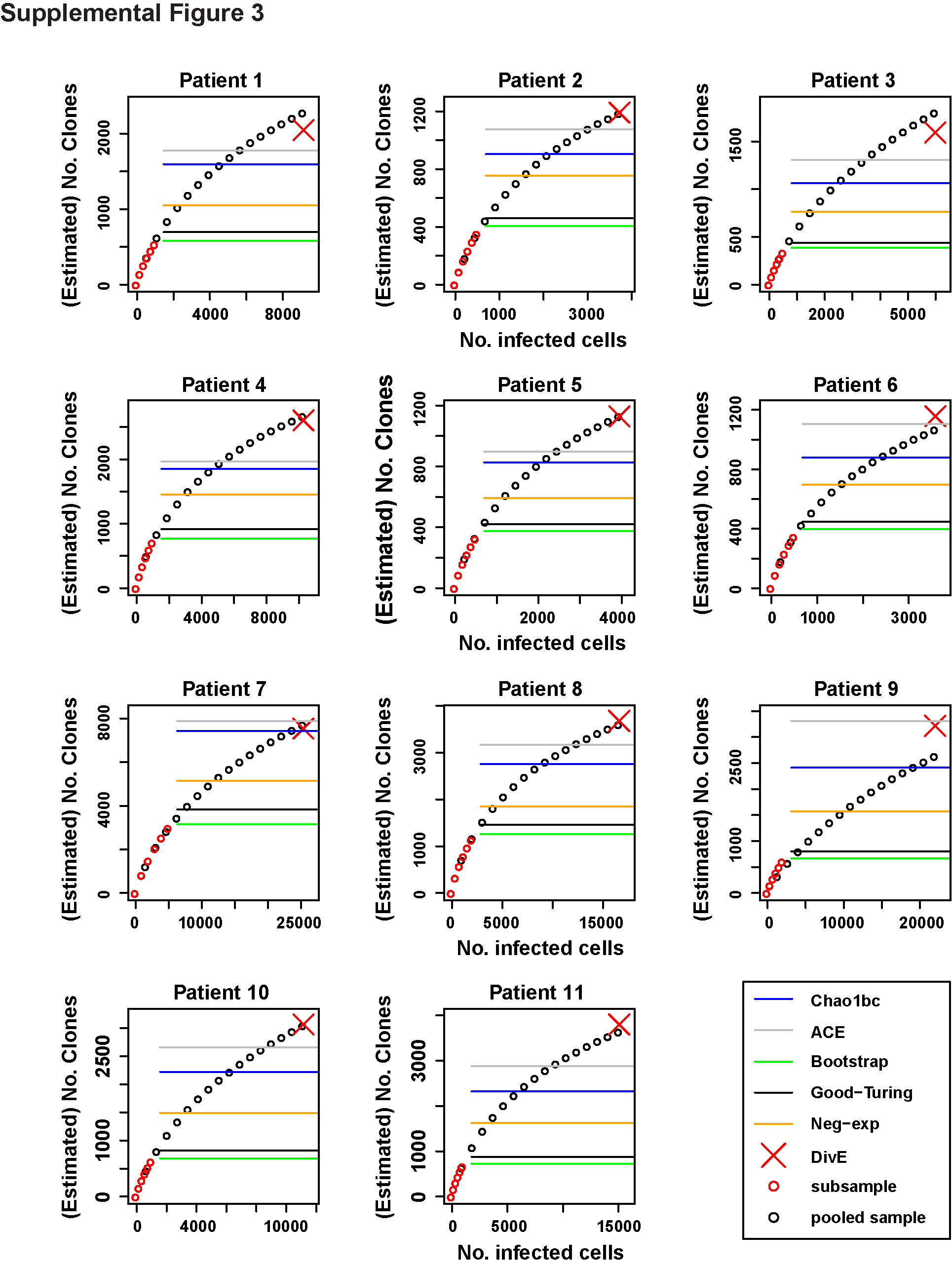

Supplement: Figure S3 — Existing estimators underestimate diversity in HTLV-1 infection. As for Figure 5. For each patient, three independent samples are pooled. Rarefaction curves from the pooled sample (black circles) and a subsample (red circles) are shown. Chao1bc, ACE, Bootstrap, Good-Turing and negative exponential estimates (blue, grey, green, black, and orange lines respectively) from the subsample, and DivE estimates (red cross) from the same subsample are plotted. All estimators except DivE typically estimate fewer clones than observed in pooled sample. In contrast, DivE accurately estimates the pooled sample species richness from the subsample. (TIF) [file pcbi.1003646.s003.tif]

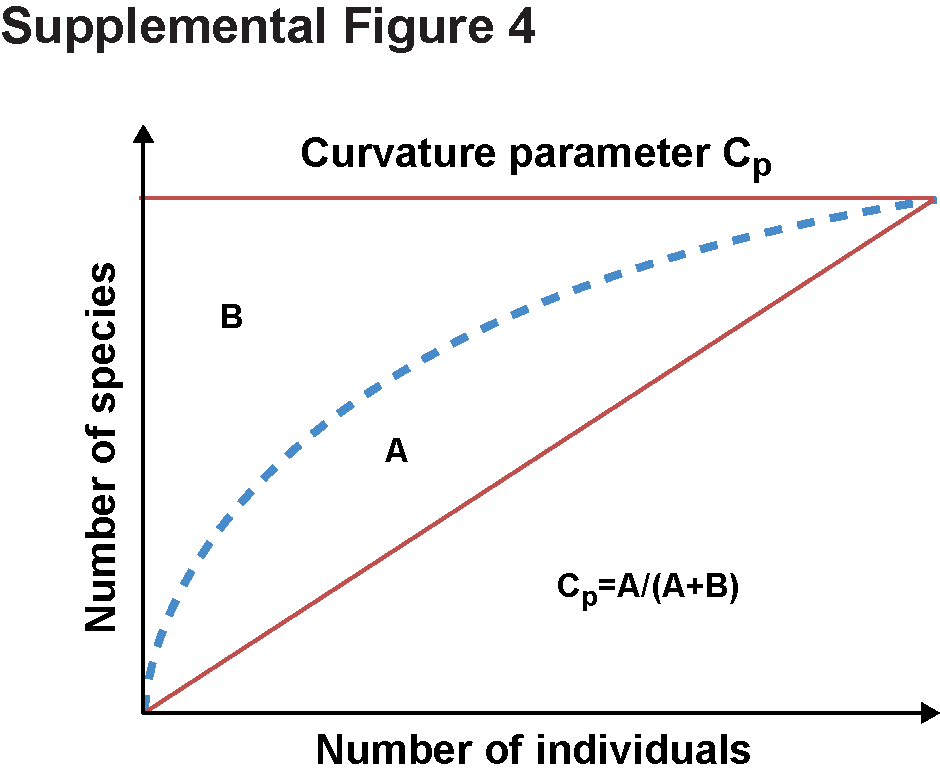

Supplement: Figure S4 — Rarefaction curvature parameter Cp . Rarefaction curves (dashed) and lines of constant rate of species-accumulation and perfect saturation (solid) are shown. Areas between the line of constant rate of species-accumulation and the rarefaction curve (A), and between the rarefaction curve and the line of perfect saturation (B) are indicated. Note Cp = 0 when the rarefaction curve is linear. (TIF) [file pcbi.1003646.s004.tif]

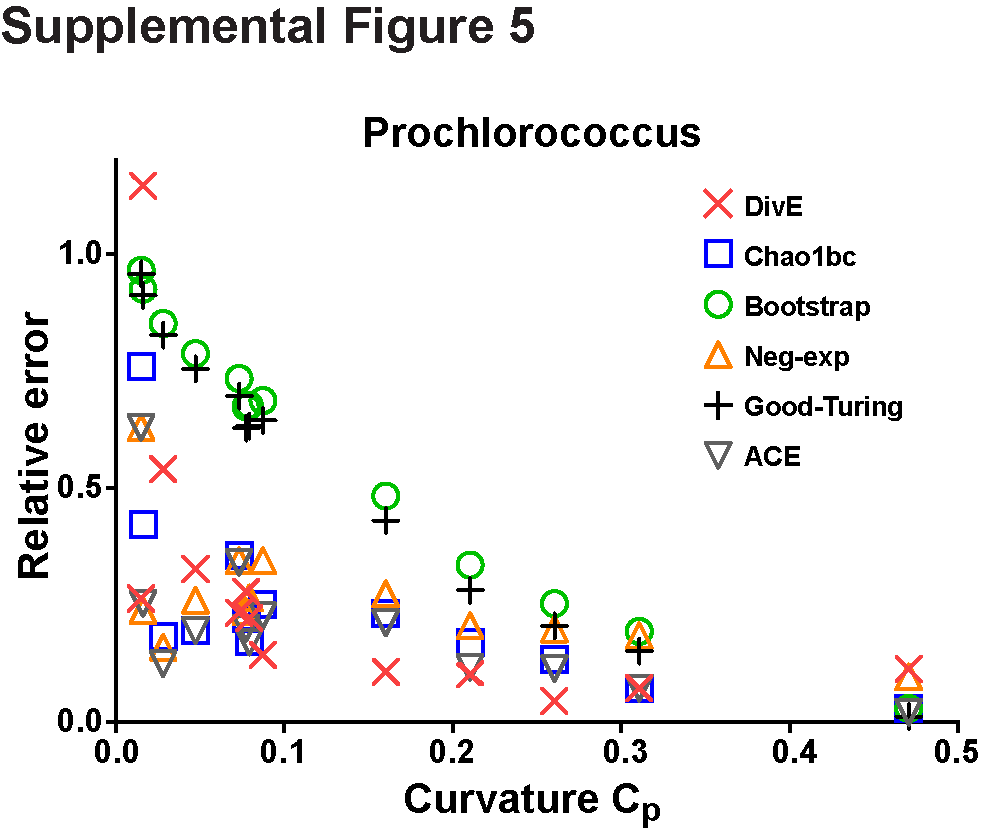

Supplement: Figure S5 — Performance of species richness estimators in metagenomic data. The curvature parameter Cp is plotted against the relative error (|Sobs - Ŝobs| /Sobs) of each estimator. Each point represents an estimate from a sample from the Prochlorococcus data. As with the TCR data, DivE typically outperforms the other estimators from Cp≈0.1 onwards. As predicted, DivE is prone to error at lower values of Cp, but becomes more accurate as Cp increases. (TIF) [file pcbi.1003646.s005.tif]

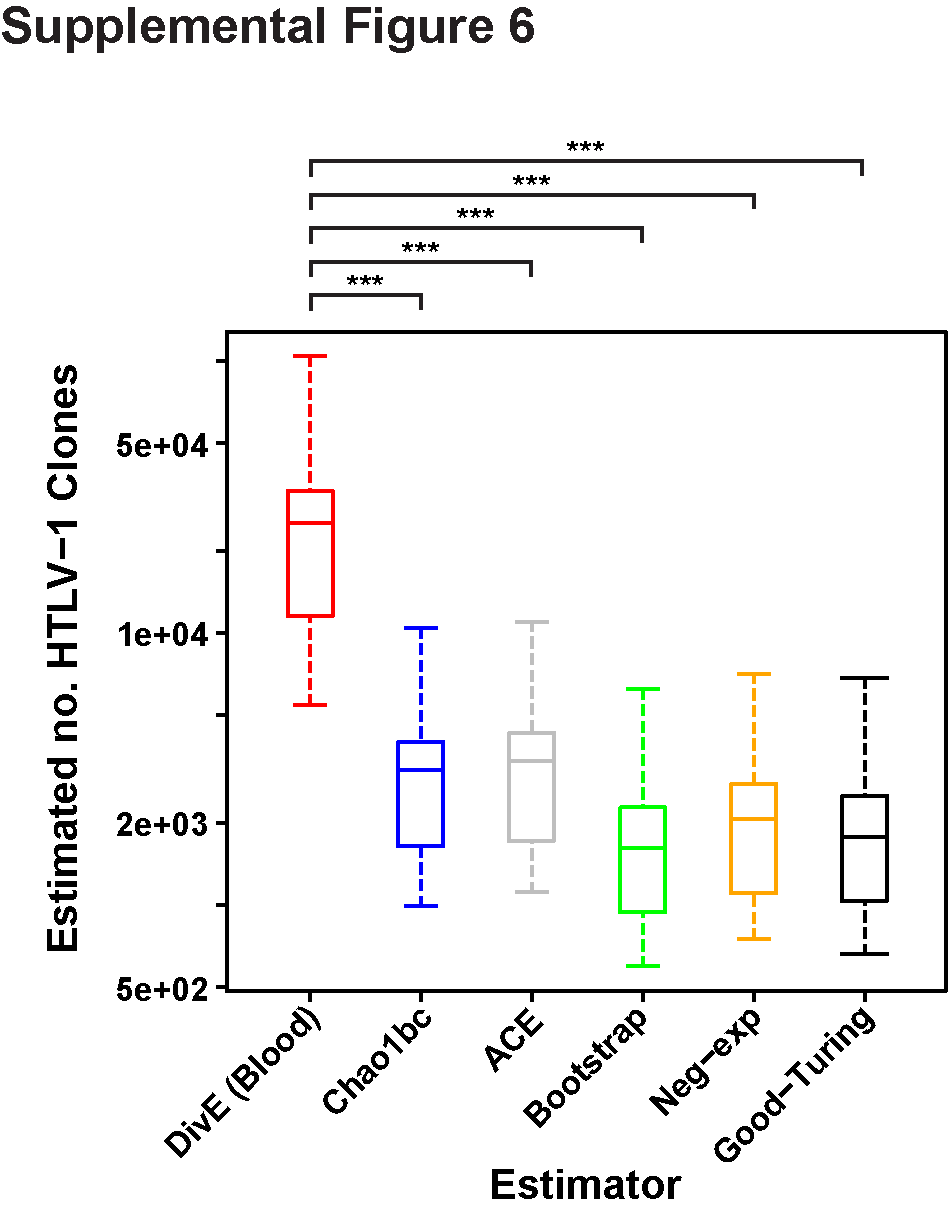

Supplement: Figure S6 — Diversity estimates in HTLV-1 infection by estimator. Each estimator was applied to 105 patient datasets, from 14 different HTLV-1+ subjects. All subjects either had HTLV-1-associated myelopathy/tropical spastic paraparesis or were asymptomatic. (TIF) [file pcbi.1003646.s006.tif]

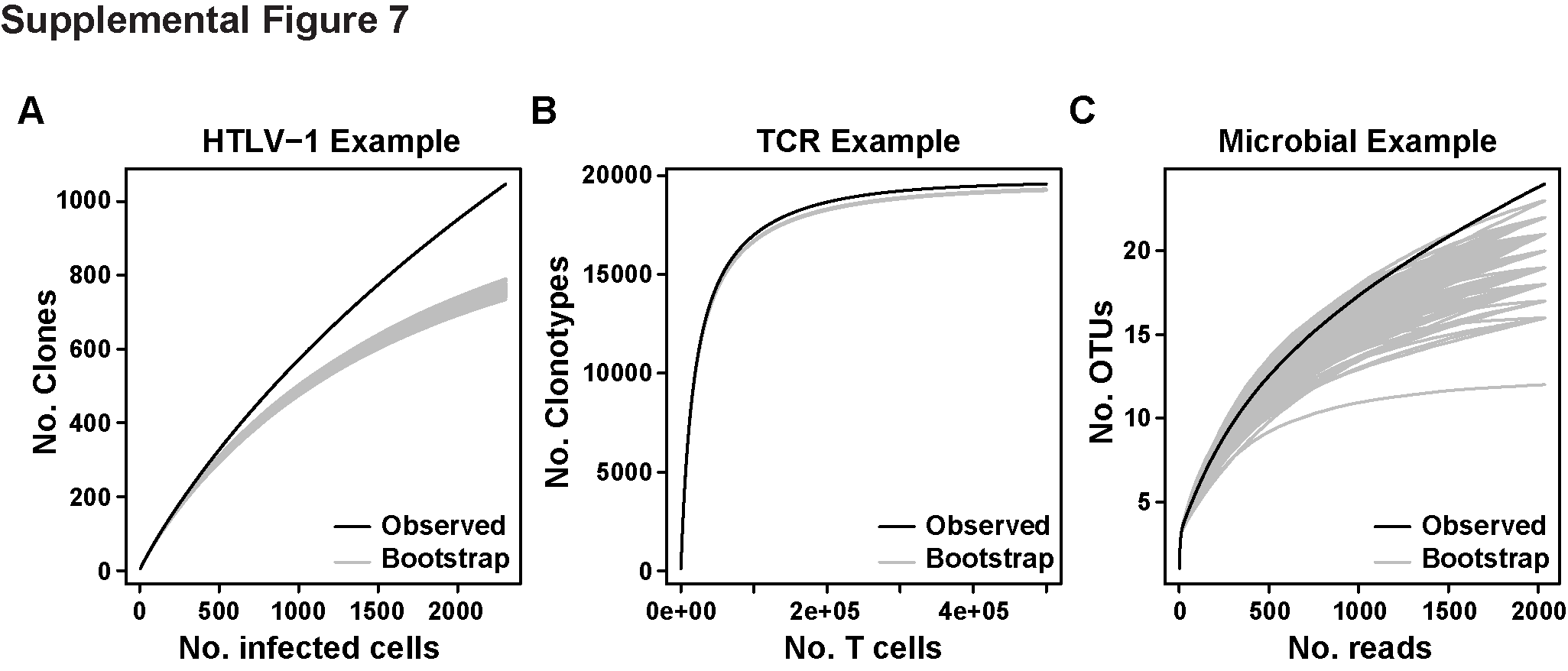

Supplement: Figure S7 — Rarefaction plots from bootstrap samples of HTLV-1, TCR, and microbial data. Rarefaction plots from 100 bootstrap samples (grey) for each of A HTLV-1, B TCR, and C microbial data. The species richness of the bootstrap samples is at most the species richness of the original data (black), and is substantially less in the majority of cases, although this effect is less noticeable with the TCR data. (TIF) [file pcbi.1003646.s007.tif]
