# Supplementary material for: Quantification of HTLV-1 Clonality and TCR Diversity
Source: PLoS Comput Biol. 2014 Jun 19;10(6):e1003646. doi: 10.1371/journal.pcbi.1003646 (PMC4063693; doi:10.1371/journal.pcbi.1003646)
Supplement: Table S1 — Subsamples used in analysis of relationship between sample size and estimated diversity, and in comparison of DivE with AICc. 1 Where there were multiple samples at multiple time points in a given HTLV-1-infected subject, a single sample at a single time point was chosen at random. (PDF) [file pcbi.1003646.s008.pdf]

Table S1. Subsamples used in analysis of relationship between sample size and estimated diversity, and in comparison of *DivE* with AIC_c.

Data Source	Patient[1]	Population/ Phenotype	Subsample Sizes	Subsample Percentages
HTLV-1	H1	NA	500, 1000, 2000, 3000, 3992	12.5, 25.1, 50.1, 75.2, 100
HTLV-1	H2	NA	500, 1000, 1623	30.8, 61.6, 100
HTLV-1	H3	NA	500, 1000, 2000, 2002	25, 50, 99.9, 100
HTLV-1	H4	NA	500, 1000, 2000, 3000, 3406	14.7, 29.4, 58.7, 88.1, 100
HTLV-1	H5	NA	500, 1000, 1693	29.5, 59.1, 100
HTLV-1	H6	NA	500, 1000, 1127	44.4, 88.7, 100
HTLV-1	H7	NA	500, 1000, 5000, 9000, 12962	3.86, 7.71, 38.6, 69.4, 100
HTLV-1	H8	NA	500, 1000, 2000, 4000, 5388	9.28, 18.6, 37.1, 74.2, 100
HTLV-1	H9	NA	500, 1000, 2000, 4000, 5007	9.99, 20, 39.9, 79.9, 100
HTLV-1	H10	NA	500, 1000, 2000, 3000, 3716	13.5, 26.9, 53.8, 80.7, 100
HTLV-1	H11	NA	500, 1000, 2000, 3000, 3907	12.8, 25.6, 51.2, 76.8, 100
HTLV-1	H12	NA	500, 1000, 2000, 3000, 4596	10.9, 21.8, 43.5, 65.3, 100
HTLV-1	H13	NA	500, 1000, 2000, 3000, 4210	11.9, 23.8, 47.5, 71.3, 100
HTLV-1	H14	NA	500, 1000, 2000, 4000, 4967	10.1, 20.1, 40.3, 80.5, 100
TCR	T1	CD4 Total	166667, 333333, 5e+05, 666667, 833333, 1e+06	16.7, 33.3, 50, 66.7, 83.3, 100
TCR	T2	CD4 Total	333333, 666667, 1e+06, 1333333, 1666667, 2e+06	16.7, 33.3, 50, 66.7, 83.3, 100
TCR	T1	CD8 Total	116667, 233333, 350000, 466667, 583333, 7e+05	16.7, 33.3, 50, 66.7, 83.3, 100
TCR	T2	CD8 Total	166667, 333333, 5e+05, 666667, 833333, 1e+06	16.7, 33.3, 50, 66.7, 83.3, 100
TCR	T1	CD4 Naïve	83333, 166667, 250000, 333333, 416667, 5e+05	16.7, 33.3, 50, 66.7, 83.3, 100
TCR	T1	CD4 CM	83333, 166667, 250000, 333333, 416667, 5e+05	16.7, 33.3, 50, 66.7, 83.3, 100
TCR	T1	CD4 EM	21667, 43333, 65000, 86667, 108333, 130000	16.7, 33.3, 50, 66.7, 83.3, 100
TCR	T1	CD8 Naïve	8167, 16333, 24500, 32667, 40833, 49000	16.7, 33.3, 50, 66.7, 83.3, 100
TCR	T1	CD8 CM	12667, 25333, 38000, 50667, 63333, 76000	16.7, 33.3, 50, 66.7, 83.3, 100
TCR	T1	CD8 EM	2833, 5667, 8500, 11333, 14167, 17000	16.7, 33.3, 50, 66.7, 83.3, 100
TCR	T2	CD4 Naïve	107833, 215667, 323500, 431333, 539167, 647000	16.7, 33.3, 50, 66.7, 83.3, 100
TCR	T2	CD4 EM	35000, 70000, 105000, 140000, 175000, 210000	16.7, 33.3, 50, 66.7, 83.3, 100
TCR	T2	CD4 CM	85500, 171000, 256500, 342000, 427500, 513000	16.7, 33.3, 50, 66.7, 83.3, 100
TCR	T2	CD8 Naïve	42667, 85333, 128000, 170667, 213333, 256000	16.7, 33.3, 50, 66.7, 83.3, 100
TCR	T2	CD8 EM	6783, 13567, 20350, 27133, 33917, 40700	16.7, 33.3, 50, 66.7, 83.3, 100
TCR	T2	CD8 CM	5667, 11333, 17000, 22667, 28333, 34000	16.7, 33.3, 50, 66.7, 83.3, 100
Microbial	M1	Primer 1	427, 640, 853, 1067, 1280	33.4, 50, 66.6, 83.4, 100
Microbial	M2	Primer 1	689, 1034, 1378, 1722, 2067	33.3, 50, 66.7, 83.3, 100
Microbial	M3	Primer 1	685, 1028, 1371, 1713, 2056	33.3, 50, 66.7, 83.3, 100
Microbial	M4	Primer 1	720, 1080, 1439, 1799, 2159	33.3, 50, 66.7, 83.3, 100
Microbial	M5	Primer 1	679, 1018, 1358, 1698, 2037	33.3, 50, 66.7, 83.4, 100
Microbial	M6	Primer 1	687, 1031, 1375, 1718, 2062	33.3, 50, 66.7, 83.3, 100
Microbial	M7	Primer 1	820, 1230, 1639, 2049, 2459	33.3, 50, 66.7, 83.3, 100
Microbial	M8	Primer 1	774, 1161, 1548, 1935, 2322	33.3, 50, 66.7, 83.3, 100
Microbial	M9	Primer 1	786, 1178, 1571, 1964, 2357	33.3, 50, 66.7, 83.3, 100
Microbial	M10	Primer 1	789, 1184, 1579, 1973, 2368	33.3, 50, 66.7, 83.3, 100
Microbial	M1	Primer 2	381, 571, 761, 952, 1142	33.4, 50, 66.6, 83.4, 100
Microbial	M2	Primer 2	522, 782, 1043, 1304, 1565	33.4, 50, 66.6, 83.3, 100
Microbial	M3	Primer 2	567, 850, 1133, 1417, 1700	33.4, 50, 66.6, 83.4, 100
Microbial	M4	Primer 2	503, 755, 1007, 1258, 1510	33.3, 50, 66.7, 83.3, 100
Microbial	M5	Primer 2	568, 852, 1135, 1419, 1703	33.4, 50, 66.6, 83.3, 100
Microbial	M6	Primer 2	640, 960, 1281, 1601, 1921	33.3, 50, 66.7, 83.3, 100
Microbial	M7	Primer 2	698, 1046, 1395, 1744, 2093	33.3, 50, 66.7, 83.3, 100

Microbial	M8	Primer 2	589, 884, 1179, 1473, 1768	33.3, 50, 66.7, 83.3, 100
Microbial	M9	Primer 2	531, 797, 1063, 1328, 1594	33.3, 50, 66.7, 83.3, 100
Microbial	M10	Primer 2	605, 907, 1209, 1512, 1814	33.4, 50, 66.6, 83.4, 100

[1] Where there were multiple samples at multiple time points in a given HTLV-1-infected subject, a single sample at a single time point was chosen at random.
