# Supplementary material for: Quantification of HTLV-1 Clonality and TCR Diversity
Source: PLoS Comput Biol. 2014 Jun 19;10(6):e1003646. doi: 10.1371/journal.pcbi.1003646 (PMC4063693; doi:10.1371/journal.pcbi.1003646)
Supplement: Table S2 — Comparison of estimates produced by DivE and by weighted, second order Akaike's Information Criterion ( AICc ). 1 Average percentage error between Sobs and Ŝobs for small subsamples for each data source. Small subsamples were defined as those ≤50% of the size of each patient data set. 2 Large subsamples defined as those >50% of the size of each patient data set. 3 Average percentage error between Sobs and Ŝobs across all patient datasets and subsamples for each data source error. (PDF) [file pcbi.1003646.s009.pdf]

Table S2. Comparison of estimates produced by *DivE* and by weighted, second order Akaike's Information Criterion (AIC_c)

Data source	Method	Error in Small Subsamples[1] (%)	Error in Large Subsamples[2] (%)	Overall Error[3] (%)
Collated	AIC_c	11.3	1.9	5.0
	DivE	5.5	1.4	2.7
HTLV-1	AIC_c	13.1	0.5	6.7
	DivE	6.2	0.5	3.3
TCR	AIC_c	2.4	0.5	1.1
	DivE	1.8	0.6	1.0
Microbial	AIC_c	23.0	3.7	7.5
	DivE	10.4	2.4	4.0

[1] Average percentage error between S_{obs} and $\hat{S}_{obs}$ for small subsamples for each data source. Small subsamples were defined as those ≤ 50% of the size of each patient data set.

[2] Large subsamples defined as those > 50% of the size of each patient data set.

[3] Average percentage error between S_{obs} and $\hat{S}_{obs}$ across all patient datasets and subsamples for each data source
