# Supplementary material for: Quantification of HTLV-1 Clonality and TCR Diversity
Source: PLoS Comput Biol. 2014 Jun 19;10(6):e1003646. doi: 10.1371/journal.pcbi.1003646 (PMC4063693; doi:10.1371/journal.pcbi.1003646)
Supplement: Table S3 — Estimator error variation with curvature in TCR data. * Median absolute percentage error between Sobs and Ŝobs. † Low curvatures Cp in range 0.016≤Cp≤0.101, intermediate curvatures in range 0.11≤Cp≤0.62. ‡ p-value of the significance of the differences between the errors of DivE and each other estimator, for each curvature range. (PDF) [file pcbi.1003646.s010.pdf]

Table S3. Estimator error variation with curvature in TCR data

Estimator	Median Error * (low C_p)†	P-value ‡	Median Error (intermediate C_p) †	P-value ‡	Median Error (all subsamples)	P-value ‡
Chao1bc	56.9	0.45	42.3	0.002	46.8	0.0015
ACE	55.0	0.45	44.2	0.002	46.6	0.0015
Bootstrap	91.4	0.45	53.0	<0.0001	72.1	<0.0001
Negative-exponential	60.2	0.45	51.4	0.0002	53.9	0.0003
Good-Turing	90.0	0.45	51.4	<0.0001	69.0	< 0.0001
DivE	23.1	NA	6.5		7.9	NA

* Median absolute percentage error between S_{obs} and $\hat{S}_{obs}$

† Low curvatures C_p in range $0.016 \leq C_p \leq 0.101$, intermediate curvatures in range $0.11 \leq C_p \leq 0.62$

‡ p-value of the significance of the differences between the errors of *DivE* and each other estimator, for each curvature range.
