# Supplementary material for: Quantification of HTLV-1 Clonality and TCR Diversity
Source: PLoS Comput Biol. 2014 Jun 19;10(6):e1003646. doi: 10.1371/journal.pcbi.1003646 (PMC4063693; doi:10.1371/journal.pcbi.1003646)
Supplement: Table S4 — DivE species richness estimates for HTLV-1 data. (PDF) [file pcbi.1003646.s011.pdf]

## Table S4. *DivE* species richness estimates for HTLV-1 data

| Patient | Timepoint | *DivE* | *DivE* lower bound | *DivE* upper bound |
|---|---|---|---|---|
| 1 | 1 | 2.5E+04 | 3.2E+03 | 1.7E+06 |
| 1 | 1 | 2.7E+04 | 3.5E+03 | 2.0E+06 |
| 1 | 1 | 2.4E+04 | 3.1E+03 | 1.6E+06 |
| 1 | 2 | 2.5E+04 | 3.4E+03 | 1.3E+06 |
| 1 | 2 | 2.6E+04 | 3.7E+03 | 1.2E+06 |
| 1 | 2 | 1.8E+04 | 3.0E+03 | 6.3E+05 |
| 1 | 3 | 2.4E+04 | 3.5E+03 | 1.1E+06 |
| 1 | 4 | 1.8E+04 | 3.2E+03 | 5.0E+05 |
| 1 | 4 | 2.5E+04 | 3.0E+03 | 2.0E+06 |
| 1 | 4 | 2.0E+04 | 3.0E+03 | 7.5E+05 |
| 2 | 1 | 1.0E+04 | 2.0E+03 | 1.9E+05 |
| 2 | 1 | 8.8E+03 | 1.8E+03 | 1.5E+05 |
| 2 | 1 | 1.1E+04 | 1.8E+03 | 3.0E+05 |
| 2 | 2 | 1.1E+04 | 1.8E+03 | 3.4E+05 |
| 2 | 2 | 9.5E+03 | 2.0E+03 | 1.5E+05 |
| 2 | 2 | 1.2E+04 | 2.0E+03 | 3.3E+05 |
| 2 | 3 | 6.4E+03 | 1.6E+03 | 1.2E+05 |
| 2 | 3 | 9.0E+03 | 1.6E+03 | 2.5E+05 |
| 2 | 3 | 9.0E+03 | 1.6E+03 | 2.3E+05 |
| 3 | 1 | 2.6E+04 | 3.4E+03 | 1.3E+06 |
| 3 | 1 | 3.3E+04 | 3.6E+03 | 2.0E+06 |
| 3 | 1 | 3.2E+04 | 3.8E+03 | 1.5E+06 |
| 3 | 2 | 3.1E+04 | 3.9E+03 | 1.5E+06 |
| 3 | 2 | 2.8E+04 | 3.8E+03 | 1.1E+06 |
| 3 | 2 | 2.5E+04 | 3.3E+03 | 1.5E+06 |
| 3 | 3 | 1.9E+04 | 2.7E+03 | 9.7E+05 |
| 3 | 3 | 2.4E+04 | 3.0E+03 | 1.2E+06 |
| 3 | 3 | 2.1E+04 | 2.8E+03 | 9.8E+05 |
| 4 | 1 | 2.0E+04 | 3.6E+03 | 3.9E+05 |
| 4 | 1 | 1.9E+04 | 3.5E+03 | 3.7E+05 |
| 4 | 1 | 1.7E+04 | 3.5E+03 | 2.5E+05 |
| 4 | 2 | 2.0E+04 | 3.8E+03 | 3.7E+05 |
| 4 | 2 | 1.9E+04 | 4.3E+03 | 2.3E+05 |
| 4 | 2 | 2.4E+04 | 4.1E+03 | 5.5E+05 |
| 4 | 3 | 2.1E+04 | 3.9E+03 | 4.4E+05 |
| 4 | 3 | 1.7E+04 | 3.6E+03 | 2.2E+05 |
| 4 | 3 | 2.3E+04 | 4.0E+03 | 5.6E+05 |
| 5 | 1 | 8.9E+03 | 1.6E+03 | 2.1E+05 |
| 5 | 1 | 1.1E+04 | 1.9E+03 | 2.7E+05 |
| 5 | 1 | 9.8E+03 | 1.8E+03 | 2.2E+05 |
| 5 | 2 | 7.3E+03 | 1.6E+03 | 1.1E+05 |
| 5 | 2 | 8.2E+03 | 1.7E+03 | 1.4E+05 |
| 5 | 2 | 7.4E+03 | 1.6E+03 | 1.7E+05 |

| | | | | |
|---|---|---|---|---|
| 5 | 3 | 8.7E+03 | 1.6E+03 | 2.6E+05 |
| 5 | 3 | 8.1E+03 | 1.5E+03 | 2.0E+05 |
| 5 | 3 | 7.2E+03 | 1.5E+03 | 1.7E+05 |
| 6 | 1 | 1.1E+04 | 2.1E+03 | 2.2E+05 |
| 6 | 1 | 8.8E+03 | 1.8E+03 | 1.4E+05 |
| 6 | 1 | 1.1E+04 | 2.1E+03 | 1.9E+05 |
| 6 | 2 | 7.4E+03 | 1.8E+03 | 6.7E+04 |
| 6 | 2 | 1.1E+04 | 2.0E+03 | 1.8E+05 |
| 6 | 2 | 8.6E+03 | 1.7E+03 | 1.1E+05 |
| 6 | 3 | 5.7E+03 | 1.5E+03 | 4.7E+04 |
| 6 | 3 | 9.0E+03 | 1.7E+03 | 1.9E+05 |
| 6 | 3 | 5.5E+03 | 1.5E+03 | 3.8E+04 |
| 7 | 1 | 7.7E+04 | 9.7E+03 | 4.7E+06 |
| 7 | 1 | 7.7E+04 | 9.2E+03 | 5.1E+06 |
| 7 | 1 | 8.1E+04 | 1.0E+04 | 5.1E+06 |
| 7 | 2 | 7.1E+04 | 9.9E+03 | 3.2E+06 |
| 7 | 2 | 6.7E+04 | 9.9E+03 | 1.9E+06 |
| 7 | 2 | 7.4E+04 | 9.0E+03 | 4.5E+06 |
| 7 | 3 | 6.8E+04 | 1.0E+04 | 2.3E+06 |
| 7 | 3 | 6.5E+04 | 1.0E+04 | 2.3E+06 |
| 7 | 3 | 7.8E+04 | 9.7E+03 | 4.7E+06 |
| 8 | 1 | 3.5E+04 | 5.0E+03 | 1.4E+06 |
| 8 | 1 | 3.0E+04 | 5.2E+03 | 7.4E+05 |
| 8 | 1 | 3.0E+04 | 5.8E+03 | 5.2E+05 |
| 8 | 2 | 2.7E+04 | 5.2E+03 | 5.3E+05 |
| 8 | 2 | 2.3E+04 | 5.5E+03 | 2.4E+05 |
| 8 | 2 | 2.3E+04 | 5.8E+03 | 2.1E+05 |
| 8 | 3 | 2.8E+04 | 4.7E+03 | 7.2E+05 |
| 8 | 3 | 3.1E+04 | 4.5E+03 | 1.3E+06 |
| 8 | 3 | 2.6E+04 | 4.6E+03 | 6.8E+05 |
| 9 | 1 | 5.7E+04 | 4.8E+03 | 1.3E+07 |
| 9 | 1 | 5.9E+04 | 6.3E+03 | 5.3E+06 |
| 9 | 1 | 5.5E+04 | 6.2E+03 | 4.7E+06 |
| 9 | 2 | 6.8E+04 | 6.1E+03 | 9.9E+06 |
| 9 | 2 | 4.9E+04 | 7.3E+03 | 1.9E+06 |
| 9 | 2 | 6.7E+04 | 6.4E+03 | 7.7E+06 |
| 9 | 3 | 6.6E+04 | 4.5E+03 | 2.7E+07 |
| 9 | 3 | 5.3E+04 | 4.3E+03 | 1.0E+07 |
| 9 | 3 | 5.5E+04 | 4.6E+03 | 1.0E+07 |
| 10 | 1 | 3.3E+04 | 4.5E+03 | 1.8E+06 |
| 10 | 1 | 3.9E+04 | 4.8E+03 | 2.5E+06 |
| 10 | 1 | 3.8E+04 | 4.7E+03 | 2.5E+06 |
| 10 | 2 | 3.0E+04 | 5.0E+03 | 8.8E+05 |
| 10 | 2 | 3.2E+04 | 5.3E+03 | 8.2E+05 |
| 10 | 2 | 3.3E+04 | 4.9E+03 | 1.2E+06 |
| 10 | 3 | 3.0E+04 | 4.0E+03 | 1.6E+06 |

| | | | | |
|---|---|---|---|---|
| 10 | 3 | 3.2E+04 | 4.8E+03 | 1.2E+06 |
| 10 | 3 | 2.8E+04 | 4.2E+03 | 9.9E+05 |
| 11 | 1 | 2.2E+04 | 3.7E+03 | 6.9E+05 |
| 11 | 1 | 3.1E+04 | 4.3E+03 | 1.4E+06 |
| 11 | 1 | 3.6E+04 | 5.1E+03 | 1.6E+06 |
| 11 | 2 | 2.7E+04 | 4.7E+03 | 7.2E+05 |
| 11 | 2 | 2.6E+04 | 5.3E+03 | 4.0E+05 |
| 11 | 2 | 3.4E+04 | 4.8E+03 | 1.5E+06 |
| 11 | 3 | 2.8E+04 | 4.9E+03 | 6.0E+05 |
| 11 | 3 | 3.2E+04 | 5.1E+03 | 9.8E+05 |
| 11 | 3 | 2.8E+04 | 5.1E+03 | 5.6E+05 |
| 12 | 1 | 1.0E+05 | 9.8E+03 | 8.3E+06 |
| 13 | 1 | 2.0E+04 | 2.9E+03 | 6.8E+05 |
| 13 | 2 | 1.6E+04 | 3.5E+03 | 1.9E+05 |
| 14 | 1 | 3.7E+04 | 3.7E+03 | 4.6E+06 |
| 14 | 2 | 2.6E+04 | 4.3E+03 | 8.1E+05 |
