# Supplementary material for: Quantification of HTLV-1 Clonality and TCR Diversity
Source: PLoS Comput Biol. 2014 Jun 19;10(6):e1003646. doi: 10.1371/journal.pcbi.1003646 (PMC4063693; doi:10.1371/journal.pcbi.1003646)
Supplement: Text S1 — Additional supporting information. (PDF) [file pcbi.1003646.s012.pdf]

Text S1. Additional supporting information.

Estimation of HTLV-1 Population Sizes

Population size is a necessary input for species richness estimation when the population must be specified, or when a saturating relationship between population size and species richness cannot be assumed. We estimate the number of HTLV-1 infected cells i) in the circulation (N_{blood}), and ii) in the whole body (N_{body}). For the former, we assumed an circulating blood volume of 5L and a peripheral blood mononuclear cell (PBMC) count of 3×10^9 L^{-1} [1]. HTLV-1 proviral load (PVL) is defined as the number of viral copies per 100 PBMCs. We assumed that each infected T cell carries a single copy of the HTLV-1 provirus [2]. Thus, we estimated that $N_{blood} = 5 \times 3 \times \text{PVL} \times 10^9$ cells.

To estimate N_{body}, we assumed that ~30% of PBMCs are $CD4^+$ T cells, and that ~95% of HTLV-1^+ cells are $CD4^+$ T cells [3]. We therefore multiplied the PVL by 100/30 to obtain the proportion of $CD4^+$ T cells that are infected, i.e. PVL_{CD4} = no. copies per 100 $CD4^+$ T cells = PVL × (100/30). The total number of lymphocytes in the body is ~2×10^{12}, and ~50% of lymphocytes are $CD4^+$ T cells. Thus N_{body} = $(100/30) \times (1/0.95) \times \text{PVL} \times 10^{12}$ HTLV-1-infected cells.

Outline of *DivE* distribution generation algorithm

Consider an observed species frequency distribution where x individuals are dispersed among species $1, \dots y$, each with observed relative frequencies $p_{x,1}, p_{x,2}, \cdots, p_{x,y}$.

$$p_x := \sum_{i=1}^{y} p_{x,i} = 1$$

Now consider the observed frequency distribution's corresponding species accumulation curve. The final point of this species accumulation data would be (x, y) (Figure 2). After fitting the models returned by *DivE* to the species accumulation curve, we extrapolate to the point $(x + a,\ y + 1)$, i.e. until we encounter another species. We determine the proportion of the $x + a$ individuals occupied by the previously observed y species. That is, we wish to calculate $p_{max} = \sum_{i=1}^{y} p_{x+a,i}$, and thus also the relative frequency of the newly observed species $y + 1$, $p_{x+a,y+1} = (1 - p_{max})$. We do this by estimating the most likely number of individuals in the wider population that belong to the species in the observed sample i.e. to species $1, \ldots, y$.

Let N be the size of the wider population from which all individuals are drawn without replacement, and let r be the number of individuals in the wider population that belong to species $1, \ldots, y$. Let d_j be the number of individuals from any of species $1, \ldots, y$ drawn on the jth trial. d_j can take values 0 or 1. Then $Pr(d_1 = 1) = {}^{r}/_{N}$, and

$$Pr(d_2 = 1 \mid d_1 = 1) = {}^{r-1}/_{N-1}$$

More generally,

$$Pr\left(d_j = 1 \mid d_{j-1} = 1 \mid \ \ldots \ \mid d_1 = 1\right) = {}^{r-(j-1)}\big/_{N-(j-1)}$$

The likelihood L of observing $(x + a - 1)$ individuals from species $1, \ldots, y$, followed by an individual belonging to a new species is thus

$$L(r) = \frac{r(r-1)(r-2)\ldots(r-(x+a-2))}{N(N-1)(N-2)\ldots(N-(x+a-2))} \frac{(N-r)}{(N-(x+a-1))}$$

where N, x and a are constants. Here, $\frac{(N-r)}{(N-(x+a-1))}$ is the probability of drawing an individual that does not belong to species $1, \ldots, y$ on the $x + a^{th}$ draw (i.e. where

$(N - (x + a - 1))$ individuals remain) when all previous draws have produced individuals belonging to species $1, \ldots, y$. Note we assume that the new species $y + 1$ is observed only once in draws $x + 1, \ldots, x + a$.

We obtain $\sum_{i=1}^{y} p_{x+a,i}$ by finding (numerically) r_{max} such that L is maximal, and compute $p_{max} = \sum_{i=1}^{y} p_{x+a,i} = {r_{max}}/{N}$.

Then for $i = 1, \ldots, y$,

$$p_{x+a,i} = p_{x,i} \times p_{max}$$
$$= p_{x,i} \times {r_{max}}/{N}$$

and

$$p_{x+a,y+1} = (1 - p_{max})$$

We now have estimates of the frequencies of species $1, \ldots, y + 1$ in $x + a$ individuals. We repeat the above process for species $y + 2$ (i.e. by extrapolation to the point $(x + a + b, y + 2)$) and so on until the predicted number of species in the population is reached (Figure 2).

REFERENCES

1. Dacie J, Lewis S (2011) Practical Haematology: Churchill Livingstone.
2. Cook LB, Rowan AG, Melamed A, Taylor GP, Bangham CRM (2012) HTLV-1-infected T cells contain a single integrated provirus in natural infection. Blood.
3. Richardson JH, Edwards AJ, Cruickshank JK, Rudge P, Dalgleish AG (1990) In vivo cellular tropism of human T-cell leukemia virus type 1. J Virol 64: 5682-5687.

List of DivE candidate models

1. Logistic

$$y = {a_1}/{(a_2 + x^{-a_3})}$$

2. Negative exponential

$$y = \left({a_1}/{a_2}\right)(1 - e^{-a_2 x})$$

3. Logarithmic

$$y = a_1 \log{(a_2 x + 1)}$$

4. Quadratic logarithmic

$$y = a_1 \log(a_3 x + 1) + a_2 \log(a_3 x + 1)^2$$

5. Quartic logarithmic

$$y = a_1 \log(a_5 x + 1) + a_2 \log(a_5 x + 1)^2 + a_3 \log(a_5 x + 1)^3 + a_4 \log(a_5 x + 1)^4$$

6. Quintic logarithmic

$$y = a_1 \log(a_6 x + 1) + a_2 \log(a_6 x + 1)^2 + a_3 \log(a_6 x + 1)^3 + a_4 \log(a_6 x + 1)^4 + a_5 \log(a_6 x + 1)^5$$

7. NIST Kirby

$$y = \frac{a_1 + a_2 x + a_3 x^2}{1 + a_4 x + a_5 x^2}$$

8. NIST MGH09

$$y = \frac{a_1(x^2 + a_2 x)}{x^2 + a_3 x + a_4}$$

9. Hyperbolic G

$$y = {a_1 x}/{(a_2 + x)} + {a_3 x}/{(a_4 + x)}$$

10. Cubic logarithmic

$$y = a_1 \log(a_4 x + 1) + a_2 \log(a_4 x + 1)^2 + a_3 \log(a_4 x + 1)^3$$

11. Gunary

$$y = {}^{x}\!/\!{}_{(a_1 + a_2 x + a_3 x^{0.5})}$$

12. Cellular Conductance

$$y = {}^{a_1}\!/\!{}_{\left(1 + e^{(x-a_2)/a_3}\right)} + {}^{a_4}\!/\!{}_{\left(1 + e^{(x-45)/a_5}\right)}$$

13. NIST Thurber

$$y = \frac{a_1 + a_2 x + a_3 x^2 + a_4 x^3}{1 + a_5 x + a_6 x^2 + a_7 x^3}$$

14. Lorentzian Modified Peak F

$$y = {}^{a_1}\!\Bigg/\!{}_{a_2 + \left({}^{(x-a_3)}\!/\!{}_{a_4}\right)^{a_5}}$$

15. Pseudo Voight peak modified

$$y = a_1 \left[a_4 \left({}^{1}\!\Big/\!{}_{\left(1 + {}^{(x-a_2)}\!/\!{}_{a_3}\right.} \right)^{a_5} + (1 - a_4) e^{-0.5\left({}^{(x-a_2)}\!/\!{}_{a_3}\right)^{a_6}} \right]$$

16. Five-parameter logistic with offset

$$y = a_1 + {}^{(a_2 - a_1)}\!\Big/\!{}_{\left(1 + \left({}^{x}\!/\!{}_{a_3}\right)^{a_4}\right)^{a_5}}$$

17. Triple exponential

$$y = a_1 e^{a_2 x} + a_3 e^{a_4 x} + a_5 e^{a_6 x} + a_7$$

18. NIST Bennett 5

$$y = a_1 \left((a_2 + x)^{\left(-1/a_3\right)} \right) + a_4$$

19. BioScience B

$$y = a_1 \left(1 - \left(1 + \left({}^{x}\!/\!{}_{a_2}\right)^{a_3}\right)^{-a_4} \right) + a_5$$

20. High-low affinity double

$$y = {}^{a_1 a_2 x}\!/_{(1 + a_2 x)} + {}^{a_3 a_4 x}\!/_{(1 + a_4 x)}$$

21. Logistic B with offset

$$y = {}^{a_1}\!\Big/_{\left(1 + \left({}^{x}\!/_{a_2}\right)^{a_3}\right)} + a_4$$

22. Hyperbolic logistic

$$y = {}^{a_1 x^{a_2}}\!\Big/_{(a_3 + x^{a_2})}$$

23. Hill

$$y = {}^{a_1 x^{a_2}}\!\Big/_{({a_3}^{a_2} + x^{a_2})}$$

24. Log-normal peak with offset

$$y = a_1 e^{\left(-0.5\left({}^{(\log(x+1) - a_2)}\!/_{a_3}\right)^2\right)} + a_4$$

25. Inverse Bleasdale

$$y = {}^{x}\!\Big/_{\left((a_1 + a_2 x)^{\left(-1/a_3\right)}\right)}$$

26. Double exponential

$$y = a_1 e^{-a_2 x} + a_3 e^{-a_4 x} + a_5$$

27. Polytrope transform with offset

$$y = {}^{a_1}\!/_{(a_3 x + a_4)^{a_2}} + a_5$$

28. Generalized product accumulation

$$y = {a_1(a_2 - x)}/{\left(a_3 + (a_2 - x)\right)} + a_4(a_2 - x) + a_5$$

29. Generalized substrate depletion

$$y = {a_1 x}/{(a_2 + x)} - a_3 x - a_4$$

30. Weibull peak

$$y = a_1 e^{\left(-0.5\left(\log({x}/{a_2})/{a_3}\right)^2\right)}$$

31. Gaussian peak modified with offset

$$y = a_1 e^{-0.5\left({(x-a_2)}/{a_3}\right)^{a_4}} + a_5$$

32. Morgan-Mercer-Flodin (MMF) with offset

$$y = {(a_1 a_2 + a_3 x^{a_4})}/{(a_2 + x^{a_4})} + a_5$$

33. Log-normal peak modified with offset

$$y = a_1 e^{\left(-0.5\left({(\log(x+1) - a_2)}/{a_3}\right)^{a_4}\right)} + a_5$$

34. Weibull

$$y = a_1 - a_2 e^{(-a_3 x^{a_4})}$$

35. Weibull peak modified with offset

$$y = a_1 - a_2 e^{\left(-0.5\left(\log({x}/{a_2})/{a_3}\right)^{a_4}\right)} + a_5$$

36. General polyfunctional 2 logs

$$y = a_1 x^{a_2} + a_3 x^{a_4} + a_5 \log(x+1)^{a_6} + a_7 \log(x+1)^{a_8}$$

37. General polyfunctional

$$y = a_1 x^{a_2} + a_3 x^{a_4} + a_5 x^{a_6} + a_7 x^{a_8}$$

38. General polyfunctional log

$$y = a_1 x^{a_2} + a_3 x^{a_4} + a_5 x^{a_6} + a_7 \log(x+1)^{a_8}$$

39. Bradley transform

$$y = a_1 \log(-a_2 \log(a_3 x + a_4))$$

40. Lorentzian modified peak C

$$y = {}^{a_1}/_{a_2} + (x - a_3)^{a_4}$$

41. Janoschek growth modified

$$y = a_1 - (a_1 - a_4)\left(1 - e^{(-a_2 x^{a_3})}\right)$$

42. General polyfunctional atan

$$y = a_1 x^{a_2} + a_3 x^{a_4} + a_5 x^{a_6} + a_7 \mathrm{atan}(x)^{a_8}$$

43. Simple equation 16

$$y = a_1 x^{(a_2 + a_3 \log(x))}$$

44. Simple equation 30

$$y = a_1 x^{(a_2 x^{a_3})}$$

45. Simple equation 26 with offset

$$y = {}^{a_1}/_{(1 + a_2 x^{a_3})^2} + a_4$$

46. Simple equation 40 with offset

$$y = a_1 \log(x + a_2)^{a_3} + a_4$$

47. Harris

$$y = {}^{1}/_{(a_1 + a_2 x^{a_3})}$$

48. General polyfunctional tanh

$$y = a_1 x^{a_2} + a_3 x^{a_4} + a_5 x^{a_6} + a_7 \tanh(x)^{a_8}$$

49. General polyfunctional negative exponential

$$y = a_1 x^{a_2} + a_3 x^{a_4} + a_5 x^{a_6} + a_7 \mathrm{e}^{-a_8 x}$$

50. General polyfunctional sin

$$y = a_1 x^{a_2} + a_3 x^{a_4} + a_5 x^{a_6} + a_7 \sin(x)^{a_8}$$

51. General polyfunctional log negative exponential

$$y = a_1 x^{a_2} + a_3 x^{a_4} + a_5 \mathrm{e}^{-a_6 x} + a_7 \log(x+1)^{a_8}$$

52. General polyfunctional log tanh

$$y = a_1 x^{a_2} + a_3 x^{a_4} + a_5 \tanh(x)^{a_6} + a_7 \log(x+1)^{a_8}$$

53. General polyfunctional log atan

$$y = a_1 x^{a_2} + a_3 x^{a_4} + a_5 \operatorname{atan}(x)^{a_6} + a_7 \log(x+1)^{a_8}$$

54. Arrhenius law stretched

$$y = a_1 e^{-\left({a_2}/{x}\right)^{a_3}}$$

55. Quadratic logarithmic variable shape

$$y = a_1 \log(a_3 x + 1) + a_2 \log(a_4 x + 1)^2$$

56. Cubic logarithmic variable shape

$$y = a_1 \log(a_4 x + 1) + a_2 \log(a_5 x + 1)^2 + a_3 \log(a_6 x + 1)^3$$

57. Quartic logarithmic variable shape

$$y = a_1 \log(a_5 x + 1) + a_2 \log(a_6 x + 1)^2 + a_3 \log(a_7 x + 1)^3 + a_4 \log(a_8 x + 1)^4$$

58. Quintic logarithmic variable shape

$$y = a_1 \log(a_6 x + 1) + a_2 \log(a_7 x + 1)^2 + a_3 \log(a_8 x + 1)^3 + a_4 \log(a_9 x + 1)^4 + a_5 \log(a_{10} x + 1)^5$$
